# Cancer metabolism in radiation sensitization – complementary roles of O-GlcNAc transferase and PARP1

**DOI:** 10.1242/jcs.264322

**Published:** 2026-03-30

**Authors:** Elena Efimova, Yue Liu, Sera Averbek, SeokGyeong Choi, Sojung Ha, Natalia Ricco, Isabelle Lomeli, Evalds Viguls, Woo-Young Kim, Stephen J. Kron

**Affiliations:** ^1^ Ludwig Center for Metastasis Research and Department of Molecular Genetics and Cell Biology, The University of Chicago, Chicago, IL 60637, USA; ^2^ College of Pharmacy, Sookmyung Women's University, Seoul 04310, Republic of Korea

**Keywords:** PARP, Radiosensitization, DNA end resection, O-GlcNAcylation, Hexosamine biosynthetic pathway, EZH2

## Abstract

For double-strand breaks (DSBs) formed by radiation, the onset of 5′ to 3′ end resection is a deciding factor in repair pathway choice, favoring homologous recombination (HR) over non-homologous end-joining (NHEJ). Studying HR-proficient MCF7 breast cancer cells, we confirmed a role for PARP1 in promoting DSB repair and limiting resection stress and identified the hexosamine biosynthetic pathway (HBP)-dependent post-translational modification O-GlcNAcylation as an independent regulator. Using pharmacological and genetic perturbations of O-linked β-N-acetylglucosamine (O-GlcNAc) transferase (OGT) and O-GlcNAcase (OGA), we showed that O-GlcNAcylation can limit end resection as measured by BrdU and RPA staining, recruitment of HR proteins BRCA1 and RAD51, and accumulation of cytosolic DNA in S/G2-phase cells. These effects were independent of PARP1 but required the histone methyltransferase EZH2. Loss of OGT or EZH2 phenocopied PARP inhibition, leading to hyper-resection after irradiation. The OGA inhibitor PUGNAc suppressed hyper-resection due to PARP1 knockout whereas treatment with the PARP inhibitor veliparib exacerbated defects in OGT- or EZH2-deficient cells. In each case, increased resection correlated with cytosolic DNA accumulation, suggesting a link to inflammatory signaling. These findings implicate the Warburg effect, via the HBP and O-GlcNAcylation, in favoring NHEJ over HR and suggest that disrupting EZH2 may sensitize HR-proficient tumor cells to radiation via resection-dependent mechanisms. Our results highlight the potential of targeting cancer-associated metabolic reprogramming to overwhelm HR repair and drive resection stress. Combining PARP inhibition with blockade of O-GlcNAcylation or EZH2 might offer a strategy to radiosensitize proliferating HR-proficient cancers while sparing non-cycling normal tissues.

## Teaser

**Summary:** PARP1 cooperates with O-GlcNAcylation and EZH2 to restrain radiation-induced DNA end resection, linking cancer metabolism to repair pathway choice and radiosensitization strategies.

## INTRODUCTION

Chromosomal DNA double-strand breaks (DSBs) are potentially lethal consequences of ionizing radiation (IR) and other genotoxic therapies. Cancer cells apply one of two major repair pathways to resolve most DSBs, choosing between error-prone re-ligation via non-homologous end joining (NHEJ) and the slower but more precise templated repair provided by homologous recombination (HR) (Nickoloff et al., 2023). Whereas NHEJ completes much of the repair within the first hours after irradiation, some breaks persist which can then fall to HR. As such, so-called DSB repair pathway choice depends in part on the complexity of the damage and on whether processing of persistent DSBs enables their repair by NHEJ. During this interval, a fraction of DSBs undergo end resection, wherein MRE11 initiates nucleolytic degradation of the 5′-strand to generate a 3′-single-stranded DNA (ssDNA) overhang (Cejka and Symington, 2021). Whereas short 3′ overhangs remain compatible with end-joining, as resection progresses from the broken end, the ssDNA can be coated with replication protein A (RPA), which can then be displaced by the recombinase RAD51. The resulting nucleoprotein filament facilitates HR repair by aligning and then recombining with an intact complementary duplex, typically a nearby sister chromatid. Multiple mechanisms restrict resection and HR to newly replicated DNA in S phase and G2, ensuring the availability of a repair template. One determinant is the competing and complementary activities of 53BP1 and BRCA1. The distinct functions of 53BP1 and BRCA1 as well as their regulation, binding partners and interactions in DSB repair pathway choice have been extensively reviewed (e.g. Ceccaldi and Cejka, 2025; Cejka and Symington, 2021; Zhao et al., 2020; Zong et al., 2025). In broad outlines, both proteins assemble at DSBs to form readily imaged foci where 53BP1 serves to protect 5′ ends while BRCA1 facilitates 5′ to 3′ resection and nucleofilament formation. 53BP1 remains active through G1/S/G2 while regulation of BRCA1 expression and function help restrict resection and HR repair to S/G2.

Disrupting DSB repair in proliferating cells has long been considered an attractive strategy to enhance the therapeutic index of radiation and other genotoxic cancer therapies (Drew et al., 2024; Groelly et al., 2023), leading to an ongoing search for targets. Recruitment and activation of poly(ADP-ribose) polymerase 1 (PARP1) is an early event in the DSB response, resulting in polymerization of NAD^+^ to form poly(ADP ribose) (PAR) chains linked to PARP1 itself, histones and other proteins at sites of DNA damage (Gupte et al., 2017). PARylation stimulates chromatin remodeling and DSB repair, with a particular impact on HR (Chaudhuri and Nussenzweig, 2017). PARP1 is commonly overexpressed in cancer cell lines (Zaremba et al., 2009) and tumors (Ossovskaya et al., 2010), making it an attractive target. Poly(ADP-ribose) polymerase inhibitors (PARPi) (Curtin and Szabo, 2020) bind the active site to interrupt this response, with many not only blocking PARP1 activity but also serving as poisons that trap PARP1 onto DNA once recruited to sites of damage (Murai et al., 2012).

Despite strong evidence for PARPi as radio- and chemo-sensitizers, combinations with radiation or chemotherapy have not progressed past clinical trials (Curtin and Szabo, 2020; Drew et al., 2024). By contrast, the selective toxicity of PARPi as single agents to cancers bearing mutations, such as loss of BRCA1 or BRCA2, that confer HR repair deficiency (HRD) has led to approvals in breast, ovarian, prostate and/or pancreatic cancers. This so-called synthetic lethality (Dibitetto et al., 2024) was initially ascribed to increased dependency on PARP1 for DNA repair and then to greater sensitivity to trapped PARP1 (Helleday, 2011) although recent studies have pointed to accumulation of replication gaps (Cong et al., 2021; Paes Dias et al., 2021) or R loops (Petropoulos et al., 2024). Beyond these defects, BRCA1, BRCA2 and PARP1 display overlapping and interdependent functions in regulation of single strand resection and nucleofilament formation for repair of DSBs. Confusingly, PARP1 has been shown to both positively and negatively influence resection and recombination via modulating multiple targets including MRE11 and BRCA1 (Chaudhuri and Nussenzweig, 2017). Caron et al. (2019) found that knockdown or knockout of PARP1 or treatment with PARPi, whether the strongly trapping PARPi talazoparib or the weakly trapping veliparib (Hopkins et al., 2015), dramatically increased end resection after irradiation. Deregulated resection might overwhelm HR repair capacity, resulting in ssDNA cleavage, accumulation of cytosolic DNA and stimulation of cGAS/STING signaling to drive anti-tumor immunity (Shen et al., 2019). The potential for hyper-resection to sensitize tumors to conventional, targeted or immunotherapy argues for further studies of PARPi in HR-proficient tumors.

**Fig. 1.**
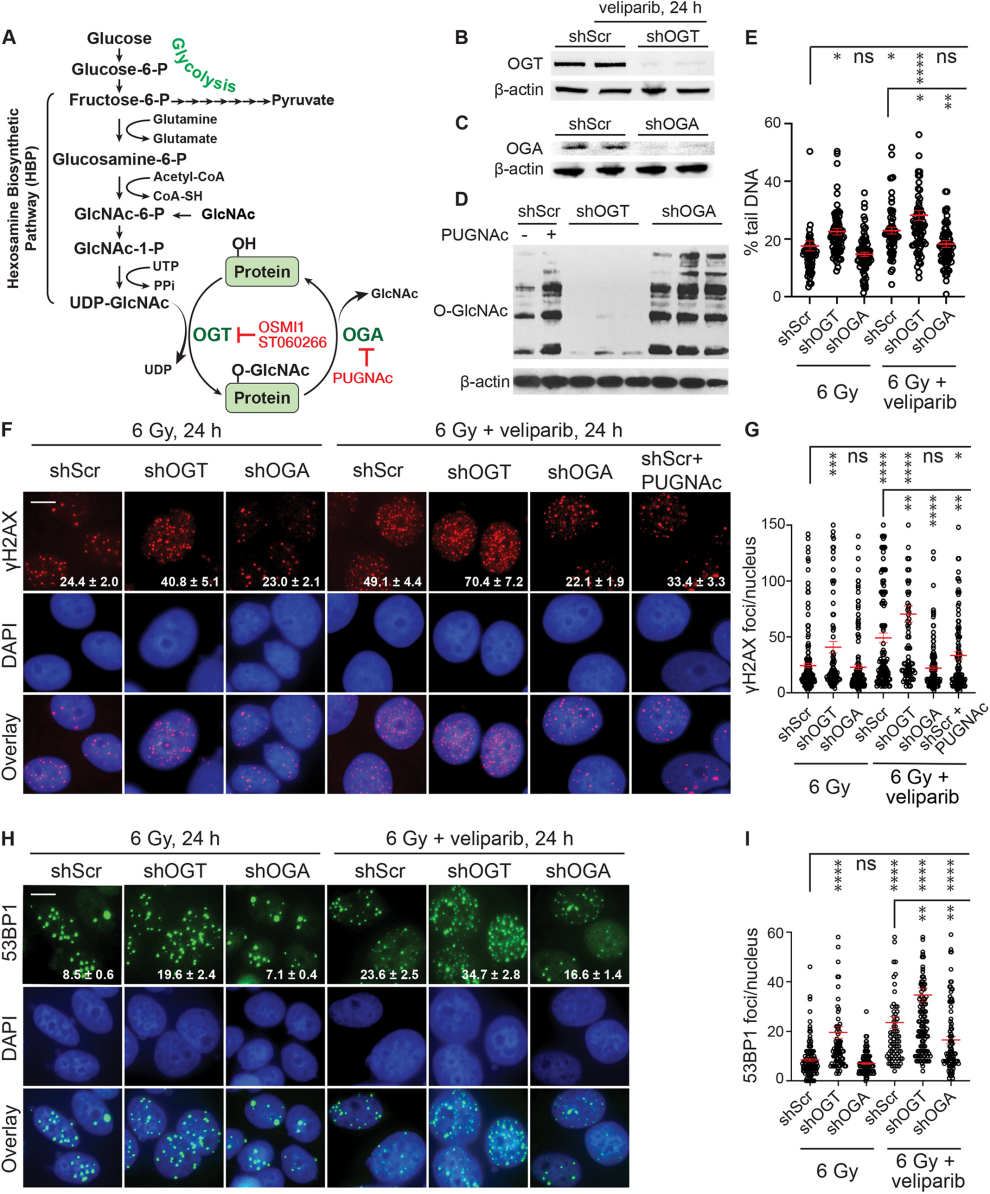
**Altering O-GlcNAcylation modulates the veliparib-induced defect in DSB repair after irradiation.** (A) The nutritional sensing HBP integrates availability and metabolism of glucose (fructose-6-phosphate, F6P), amino acids (glutamine), fatty acids (Acetyl-CoA) and nucleotides (UTP) in generating UDP-GlcNAc, a nucleotide sugar donor for protein O-GlcNAcylation. Feeding cells with GlcNAc increases UDP-GlcNAc biosynthesis without substantially changing cell metabolism. The enzyme O-GlcNAc transferase (OGT) catalyzes the addition of O-GlcNAc to serine or threonine residues of proteins, whereas O-GlcNAcase (OGA) removes this modification. OSMI-1 and ST060266 are small-molecule inhibitors of OGT, whereas PUGNAc inhibits OGA. (B,C) Western blots confirming knockdown of OGT and OGA in cells stably expressing doxycycline-inducible shRNAmir (shOGT or shOGA) compared to scrambled control (shScr) after 48 h of doxycycline (1 µg/ml) treatment. In panel B only, cells were additionally treated with 10 µM veliparib for 24 h, as indicated. The β-actin blot in B is also shown as a loading control in Fig. S1C, where the same membrane was reprobed to detect O-GlcNAcylation. (D) Western blot showing the effects of OGT and OGA knockdown on global O-GlcNAcylation. shScr, shOGT and shOGA cells were treated with 1 µg/ml doxycycline for 48 h. As a control, shScr cells were also treated overnight with 50 µM PUGNAc (an OGA inhibitor) to increase O-GlcNAcylation. For B–D, total cell lysates were analyzed, and β-actin was probed as a loading control. Blot images are representative of at least three independent repeats. (E) Compared to untargeted control (shScr), silencing OGT (shOGT) or OGA (shOGA) modulated the DSB repair defect in MCF7 cells after irradiation with or without PARP inhibitor. shRNAs were induced for 48 h with 1 µg/ml doxycycline, and cells treated for 1 h with 0 or 10 µM veliparib before 6 Gy irradiation. Neutral comet assays were performed 24 h after 6 Gy. Plots show the percentage of tail DNA for individual nuclei (circles). (F) Representative images demonstrating effects of shScr, shOGT, and shOGA at 24 h after 6 Gy with or without 10 µM veliparib on persistence of γH2AX foci (pseudo-colored in red) with DNA counterstained using DAPI (blue), with overlays. (G) Plots show the number of γH2AX foci per nucleus in individual cells as shown in F. Silencing OGT or OGA also modulates 53BP1 foci persistence in MCF7 cells after irradiation with or without PARP inhibitor. (H) Effects of shScr, shOGT, and shOGA at 24 h after 6 Gy with or without 10 µM veliparib on 53BP1 foci (pseudo-colored in green) with DAPI (blue). (I) Plots show the number of 53BP1 foci per nucleus in individual cells as shown in H. Scale bars: 20 μm; values on the images are mean±s.e.m. foci per nucleus (*n*>75). For plots, red bars indicate mean±s.e.m. (*n*>75); ****P<0.0001; ***P<0.001; **P<0.01; *P<0.05; ns, *P*>0.05 (two-tailed unpaired *t*-test).

Altered metabolism is a hallmark of cancer, with the most characteristic change being the Warburg effect, wherein cancer cells continue to take up glucose and perform glycolysis even under conditions amenable to oxidative phosphorylation (Vander Heiden et al., 2009; Warburg, 1956). Cancer metabolic reprogramming not only supports rapid proliferation by fulfilling bioenergetic and biosynthetic demands but also alters pools of metabolic intermediates that serve as cofactors in protein posttranslational modifications. Many of these can regulate DNA damage response including mono- and poly-ADP ribosylation (NAD^+^), phosphorylation (ATP), methylation [S-adenosyl methionine (SAM)], acetylation [acetyl-CoA (Ac-CoA)], lactylation (lactyl-CoA) and O-GlcNAcylation (UDP-GlcNAc, where GlcNAc is N-acetylglucosamine). Our prior work revealed a major role for the hexosamine biosynthetic pathway (HBP, Fig. 1A) in the DNA damage response. Here, the glycolysis intermediate fructose-6-phosphate (F6P) is modified with an amino group from glutamine to form glucosamine-6-phosphate (GlnN-6P) and then with an acetyl group from Ac-CoA to form GlcNAc-6P. Exchanging the phosphate and incorporation of UMP from UTP produce UDP-GlcNAc, the cofactor of O-GlcNAc transferase (OGT), the single enzyme that performs nucleocytoplasmic protein O-GlcNAcylation (Averbek et al., 2021; Efimova et al., 2019, 2016). We found that providing glucose or GlcNAc to promote UDP-GlcNAc biosynthesis and the resulting OGT-dependent protein modification or preventing O-GlcNAc hydrolysis by inhibiting O-GlcNAcase (OGA) each accelerate DSB repair in irradiated cells. In turn, limiting UDP-GlcNAc synthesis or inhibiting OGT delays DSB repair. Although multiple DNA replication and repair proteins are regulated by O-GlcNAcylation (Wu et al., 2024), we found that the methyltransferase EZH2, the catalytic subunit of polycomb repression complex II (PRC2) and the origin of the H3K27me3 repressive mark (Margueron and Reinberg, 2011), appear to mediate the effects of OGT on DSB repair. Our data implicated activation of the HBP and increased O-GlcNAcylation in promoting the initial rapid phase of rejoining. Alternatively, O-GlcNAcylation might simply appear to favor NHEJ via limiting end resection and shifting repair away from HR. This would be consistent with data that OGT modifies multiple resection proteins including BRCA1 (Averbek et al., 2021). Given that crosstalk between O-GlcNAcylation and PARP1 in DSB repair remains largely unexplored, we entertained the possibility that they similarly regulate resection and, thereby, repair pathway choice.

Here, using the HR-proficient (Mao et al., 2009) MCF7 breast cancer cell line as a model, we investigate radiosensitizing effects of modulating O-GlcNAcylation alone or in combination with inhibiting PARP1, using the low-trapping PARPi veliparib (ABT-888) (Donawho et al., 2007) as a probe. We demonstrate that targeting OGT limits DNA repair after radiation on its own and this effect is amplified in combination with PARP inhibition. Conversely, blocking OGA appears to modestly enhance DSB repair but significantly suppresses the sensitizing effects of PARP1 inhibition. PARP and OGT inhibition each lead to hyper-resection on their own, and combined inhibition results in a further increase in ssDNA formation after irradiation. The two effects appear independent insofar as O-GlcNAcylation can reduce resection in PARP1-knockout (KO) cells whereas veliparib can enhance resection in EZH2-KO cells. Importantly, targeting PARP1, OGT, EZH2 or combinations thereof resulted in accumulation of cytosolic DNA, indicating catastrophic failure of repair and providing a potential stimulus for inflammatory signaling.

The findings underscore the potential of targeting O-GlcNAcylation as a strategy to overcome intrinsic resistance due to cancer metabolic reprogramming. Inhibiting OGT or disrupting other targets in the HBP-EZH2 pathway may enhance the effectiveness of radiation and potentiate the benefits of PARP inhibition, providing a novel therapeutic strategy for HR proficient cancers.

## RESULTS

### Protein O-GlcNAcylation modulates PARP inhibitor-induced DSB repair defects

To explore how Warburg metabolism and the HBP may influence the radiosensitizing effects of the PARPi veliparib (ABT-888) (Donawho et al., 2007), we modulated O-GlcNAcylation using inducible shRNAmirs in MCF7 ER^+^ human breast cancer cells to knock down (KD) OGA or OGT (Fig. 1A–C). After 48 h treatment with doxycycline, global protein O-GlcNAcylation was increased in cells expressing shRNA targeting OGA (shOGA) and reduced in cells expressing shRNA targeting OGT (shOGT) compared to levels in non-targeted (scrambled) control shRNA (shScr) (Fig. 1D). After inducing the shRNAmirs, cells were treated with 6 Gy IR. Following 6 Gy IR, both shOGT and shOGA continued to significantly modulate global O-GlcNAcylation levels, with shOGT reducing and shOGA increasing protein O-GlcNAcylation compared to levels in shScr controls (Fig. S1A). These effects persisted 24 h after IR or veliparib treatment (Fig. S1B,C), indicating that O-GlcNAcylation regulation by shOGT and shOGA is maintained under conditions of DNA damage or PARP inhibition.

We next examined the functional impact of these changes using a neutral comet assay to evaluate levels of persistent DSBs in cells 24 h post IR (Olive and Banáth, 2006). In this assay, unrepaired DSBs increase the proportion of chromosomal DNA ends and fragments that form the comet tail (Fig. 1E). Whereas shScr cells displayed 18±2% (mean±s.e.m.) tail DNA at 24 h after IR, treatment of shScr cells for 1 h with 10 µM veliparib prior to irradiation increased tail DNA levels to 23±1% (*P*<0.01). shOGT cells displayed tail DNA levels of 23±1%, indicating reduced DSB repair, whereas shOGA cells displayed similar DSB repair to shScr cells at 15±1%. When shOGT cells were treated with veliparib, tail DNA increased to 28±2% (*P*<0.01 relative to shScr+veliparib), whereas treating shOGA cells with veliparib had little or no effect, leaving tail DNA at 18±1% (*P*<0.005 relative to shScr+veliparib).

To further explore crosstalk between PARylation and O-GlcNAcylation, we examined persistence of IR-induced DNA damage foci (IRIF) marked by γH2AX or 53BP1 at 24 h after IR (Fig. 1F–I). Formation of γH2AX foci is considered an early marker for DSBs that might subsequently be repaired by NHEJ, HR or another pathway. 53BP1 localizes to γH2AX to form foci where it serves a role in limiting end resection, favoring NHEJ over HR. In general, γH2AX and 53BP1 foci persist at DSBs that remain unrepaired (Noda et al., 2012). In non-irradiated (NIR) control cells, neither shOGT nor shOGA altered the number of 53BP1 foci compared to that seen in shScr controls, suggesting that basal DNA damage levels were not affected by O-GlcNAcylation modulation (Fig. S1D). At 24 h post IR (6 Gy), the pattern of γH2AX and 53BP1 staining mirrored the comet assay results. Veliparib treatment increased both γH2AX and 53BP1 persistence in shScr cells. shOGT cells displayed increased γH2AX and 53BP1 foci after IR, and this was further increased by veliparib treatment. Meanwhile, shOGA displayed similar levels of γH2AX and 53BP1 foci to that in shScr cells on its own but failed to display increased foci after treatment with veliparib. Chemical inhibition of OGA using PUGNAc produced similar effects to shOGA in preventing veliparib from increasing γH2AX foci in MCF7 cells. These data suggest independent and compensatory effects of PARylation and O-GlcNAcylation on DSB repair after irradiation.

### The impact of veliparib and O-GlcNAc on radiation sensitivity is specific to the cell cycle stage

**Fig. 2.**
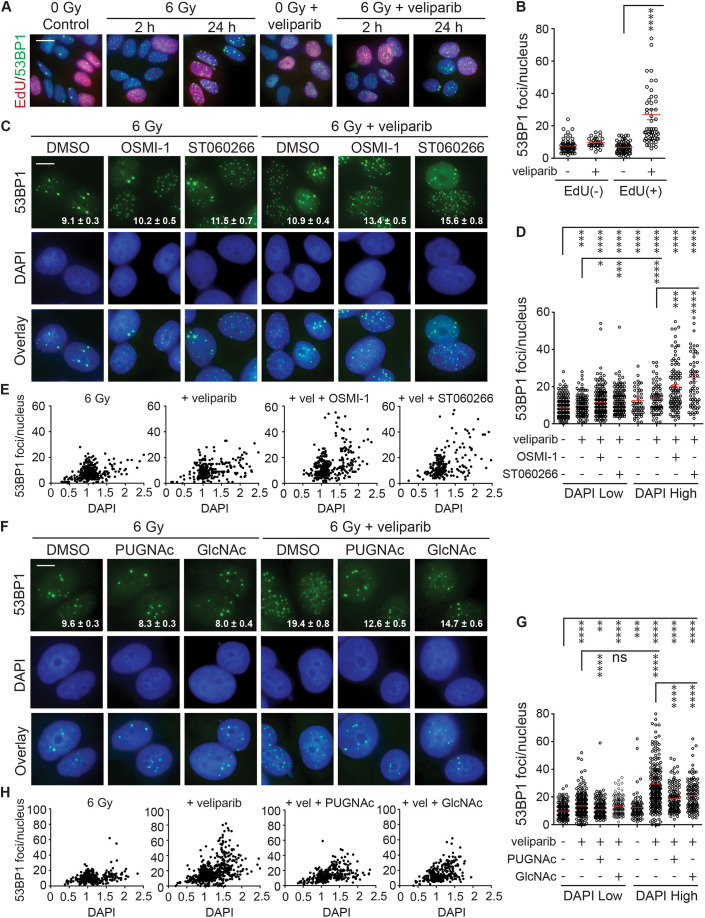
**OGT and OGA inhibitors differentially affect DNA damage foci persistence during the cell cycle in irradiated MCF7 cells.** (A) Veliparib impairs DSB repair in cells undergoing active DNA synthesis. Cycling MCF7 cells were treated with 0 or 10 μM veliparib for 1 h prior to 6 Gy. EdU was added 20 min before IR to label newly synthesized DNA during replication. Cells were then fixed at 2 and 24 h, and non-irradiated cells were fixed as controls. Immunofluorescence staining was performed to visualize 53BP1 foci and click chemistry was used to detect EdU. Shown are representative overlay images with 53BP1 in green, EdU in red, and DAPI in blue. Scale bar: 40 μm. (B) Quantification of 53BP1 foci at 24 h after 6 Gy with or without 10 µM veliparib in EdU-negative versus EdU-positive cells. (C) Inhibiting OGT promotes 53BP1 foci persistence and enhances veliparib effects. MCF7 cells were treated for 1 h before 6 Gy with DMSO control, 25 μM OSMI-1 or 2.5 μM ST060266 with or without 5 μM veliparib, irradiated with 6 Gy and fixed after 24 h. Images show 53BP1 foci (green), DAPI (blue) and overlays. Scale bars: 20 μm. (D) OGT inhibitor effects on 53BP1 foci at 24 h after 6 Gy with or without 5 µM veliparib. Cells were grouped by nuclear DAPI intensity into DAPI Low (G1 phase) and DAPI High (S/G2 phase). (E) Data from D for 6 Gy, 6 Gy+veliparib, 6 Gy+veliparib+OSMI-1, and 6 Gy+veliparib+ST060266 plotted as 53BP1 foci versus normalized DAPI intensity for individual nuclei. DAPI intensity ranges of 0.5–1.5 and 1.5–2.5 were used to define DAPI Low (presumptive G1) and DAPI High (presumptive S/G2), respectively, showing that the increase in IRIF persistence was most pronounced in S/G2. (F) Promoting O-GlcNAcylation reduces veliparib effects. MCF7 cells were treated for 1 h with DMSO, 50 μM PUGNAc or 5 mM N-acetylglucosamine (GlcNAc) with or without 10 μM veliparib, irradiated with 6 Gy and fixed after 24 h. Images show 53BP1 foci (green), DAPI (blue) and overlays. Scale bar: 20 μm. (G) O-GlcNAcylation effects on 53BP1 foci at 24 h after 6 Gy with or without 10 µM veliparib in DAPI Low and DAPI High cells. (H) Data from G for 6 Gy, 6 Gy+veliparib, 6 Gy+veliparib+PUGNAc, and 6 Gy+veliparib+GlcNAc. The same DAPI intensity ranges used in E were used to define DAPI Low and DAPI High. Values on the images are mean±s.e.m. foci per nucleus (*n*>75). For plots, red bars indicate mean±s.e.m. (*n*>75). ****P<0.0001; ***P<0.001; **P<0.01; *P<0.05; ns, *P*>0.05 (two-tailed unpaired *t*-test).

A major limitation in using formation and persistence of γH2AX and 53BP1 foci after irradiation as a proxy for DSB detection and repair is high cell-to-cell heterogeneity. This has been ascribed to altered DSB repair pathway utilization through the cell cycle. Although NHEJ remains the predominant mechanism of DSB repair throughout the cell cycle (Mao et al., 2008), HR augments end-joining repair during S and G2. Cell cycle distribution and progression are also variables, owing to G1, S or G2 checkpoint activation (Shaltiel et al., 2015). The distribution of IRIF are particularly skewed when cells are treated with veliparib and then irradiated, as in Fig. 1F,H, suggesting that PARP1 might serve a cell cycle-dependent role in DSB repair. PARP1 performs key functions during DNA replication, suggesting it has a crucial role in repair of radiation-induced DSBs in S phase. To explore which cell cycle stage is most affected by PARP inhibition, we pulsed proliferating MCF7 cells with the thymidine analog 5-ethynyl-2′-deoxyuridine (EdU) for 20 min to allow its incorporation into newly synthesized DNA, marking cells in S phase. The labeled cells were treated with veliparib and irradiated with 6 Gy, then analyzed after 2 h or 24 h by click chemistry detection of the incorporated EdU (red) and 53BP1 foci staining (green) (Fig. 2A,B). Treatment with veliparib prior to irradiation induced a modest increase in IRIF in EdU-negative cells and a more than a 4-fold increase in persistent IRIF in EdU-positive cells.

As a complementary test, we developed an MCF7 cell line stably expressing the mCherry–hCdt1 and mVenus–hGeminin fluorescent ubiquitylation-based cell cycle indicator (FUCCI) reporters (Sakaue-Sawano et al., 2008). FUCCI cell nuclei display red fluorescence in G1/G0 phase (mCherry), yellow at the G1/S transition (mCherry+mVenus), green during S/G2/M phase (mVenus) and are non-fluorescent during early G1 (Fig. S2A). We further engineered these MCF7-FUCCI cells to express 53BP1–CFP, enabling live-cell imaging of IRIF dynamics. At 24 h after 6 Gy, we observed a slight increase in 53BP1 foci persistence in cells in the S/G2 phase compared to those in the G1 phase, possibly due to the higher DNA content during S/G2 (Fig. S2B,C). Consistent with the EdU-labeling results, veliparib significantly enhanced IRIF persistence specifically in S/G2 phase cells, with minimal effects on G1 phase cells. These results suggest that veliparib predominantly affects cells in the S/G2 phase, enhancing their DNA damage foci persistence in response to IR.

Toward examining whether the effects of O-GlcNAcylation on IRIF persistence are similarly cell-cycle dependent, we pre-treated cells with the small-molecule OGT inhibitors OSMI-1 or ST060266 to reduce protein O-GlcNAcylation before treating them with veliparib and/or 6 Gy. Both OSMI-1 and ST060266 effectively reduced global protein O-GlcNAcylation without affecting baseline levels of 53BP1 foci in non-irradiated (NIR) cells (Fig. S3A–C). After 24 h post 6 Gy, we quantified persistent 53BP1 foci along with DAPI staining intensity, allowing us to separate presumptive G1-phase cells (where the low DNA content can be detected as lower DAPI intensity, DAPI Low) from S/G2-phase cells (where higher DNA content is detected as higher DAPI intensity, DAPI High) (Roukos et al., 2015; Schindelin et al., 2012). Although only modest changes in 53BP1 foci persistence were observed in DAPI Low cells, the effects of OSMI-1 and ST060266 on veliparib-induced IRIF persistence were quite pronounced in the DAPI High cells (Fig. 2C,D). Normalizing the DAPI staining to that of G1 nuclei and plotting relative DAPI intensity versus IRIF numbers confirmed only modest effects on presumptive G1 cells (DAPI intensity 0.5–1.5) but a marked increase in both the fraction of cells and IRIF persistence in S/G2 (DAPI intensity 1.5–2.5) (Fig. 2E), consistent with slow DSB repair and checkpoint delay. These results suggest that the compound effects of combined inhibition of OGT and PARP1 on DSB repair might be limited to S/G2-phase cells.

As a complementary test, we examined whether increased protein O-GlcNAcylation might resolve IRIF persistence in S/G2. Cells were pre-treated with the OGA inhibitor PUGNAc or the HBP intermediate GlcNAc, prior to treatment with veliparib and IR as described above. After 24 h, 53BP1 foci and DAPI intensity were quantified. Promoting O-GlcNAcylation with PUGNAc or GlcNAc suppressed veliparib-induced IRIF persistence in the DAPI High population (Fig. 2F,G) and apparent cell cycle delay in S/G2 (Fig. 2H).

### O-GlcNAcylation modulates recruitment and retention of HR proteins BRCA1 and RAD51

Considering that whereas NHEJ occurs throughout the cell cycle, HR is restricted to S/G2 phase, the apparent S/G2 specificity of persistent γH2AX and 53BP1 foci observed upon inhibition of OGT and/or PARP1 might be linked to a deficit in HR repair. However, insofar as 53BP1 skews DSB repair choice toward NHEJ, the connection to HR might be indirect. As direct markers for HR, we examined effects of modulating O-GlcNAcylation and/or PARylation on formation and persistence of BRCA1 and RAD51 foci.

**Fig. 3.**
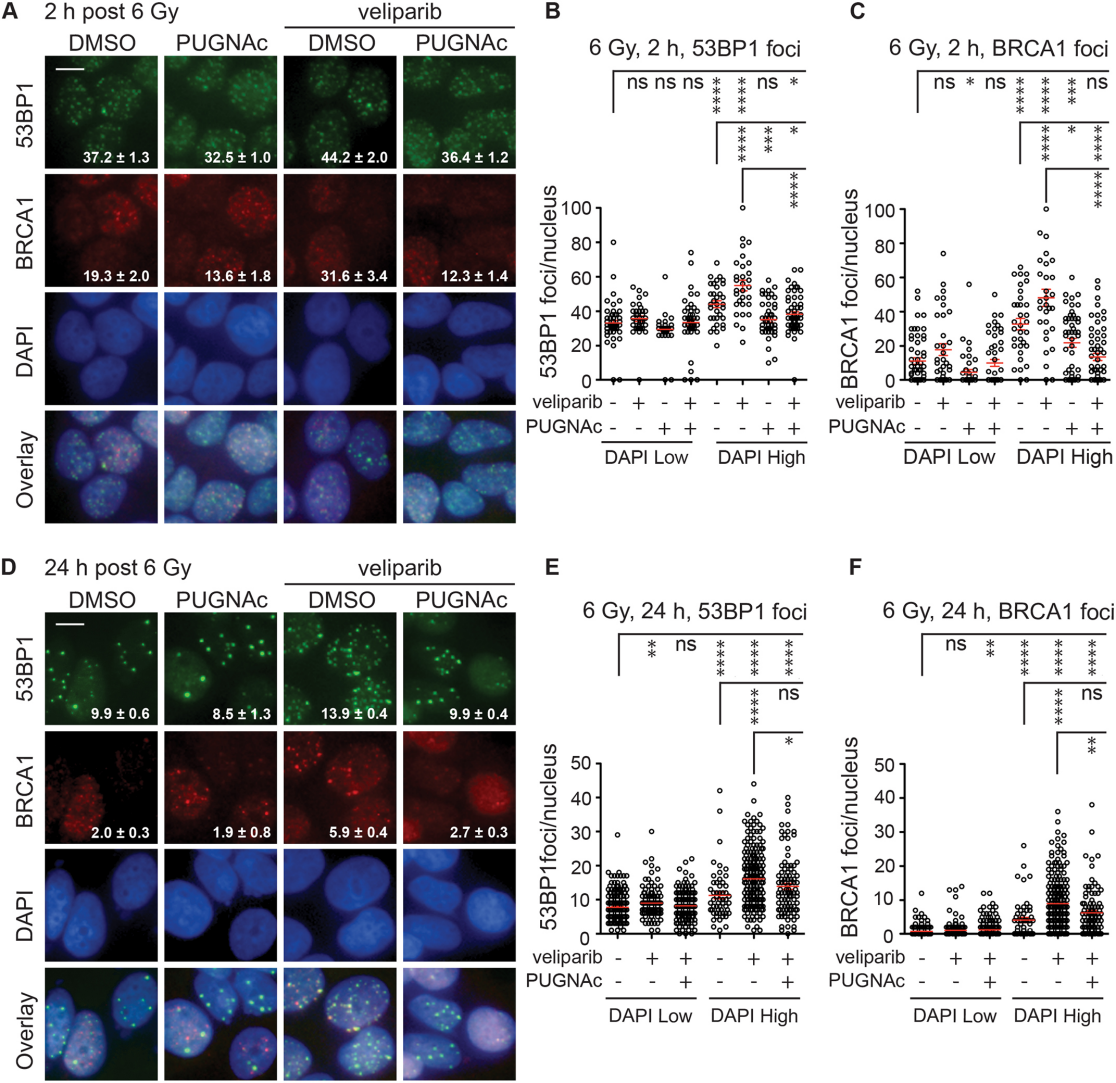
**O-GlcNAcylation antagonizes the effects of veliparib on the recruitment and retention of BRCA1 to DSBs after irradiation.** (A) MCF7 cells were treated for 1 h with DMSO or 50 μM PUGNAc with or without 10 µM veliparib, irradiated with 6 Gy, fixed after 2 h and stained for 53BP1 and BRCA1. Representative images with 53BP1 foci (green), BRCA1 foci (red), and DAPI (blue) are shown. (B,C) Plots of 53BP1 (B) and BRCA1 (C) foci per nucleus at 2 h after 6 Gy in DAPI Low and DAPI High cells. (D) Cells were treated as in A but fixed after 24 h. (E,F) Plots of 53BP1 (E) and BRCA1 (F) foci per nucleus at 24 h after 6 Gy in DAPI Low and DAPI High cells, showing that the effects of veliparib and/or PUGNAc were more pronounced in DAPI High (S/G2) cells. Scale bars: 20 μm. Values on the images are mean±s.e.m. foci per nucleus. (*n*>75). For plots, red bars indicate mean±s.e.m. (*n*>75). ****P<0.0001; ***P<0.001; **P<0.01; *P<0.05; ns, *P*>0.05 (two-tailed unpaired *t*-test).

As an initial test, cells were treated with PUGNAc and/or veliparib, irradiated with 6 Gy, fixed at 2 h (foci formation) or 24 h (foci persistence), co-stained for 53BP1 and BRCA1, and foci were quantified with DAPI staining intensity to separate G1 from S/G2 cells (Fig. 3; Fig. S4). At 2 h after 6 Gy, IRIF formation by both 53BP1 and BRCA1 was greater in S/G2 cells (DAPI High, 1.5–2.5) than G1 cells (DAPI Low, 0.5–1.5), a difference magnified by veliparib pre-treatment. Pretreatment with OGA inhibitor PUGNAc reduced IRIF formation and largely blocked the effects of veliparib for both 53BP1 and BRCA1 (Fig. 3A–C; Fig. S4A,B).

A similar pattern was observed after co-staining for 53BP1 and RAD51 at 2 h after 6 Gy (Fig. S5A–C). RAD51 foci appeared predominantly in S/G2 cells (DAPI 1.5–2.5) following IR alone, an effect increased by veliparib. Although PUGNAc co-treatment reduced the effects of veliparib on RAD51 foci, the OGT inhibitor ST060266 synergized with veliparib to further enhance RAD51 foci formation in S/G2-phase cells.

IRIF that remain persistent at 24 h after irradiation are typically more prominent and can be counted more reliably. Examining cells co-stained for 53BP1 and BRCA1 at 24 h after 6 Gy revealed a similar pattern for both markers, with marked IRIF persistence in the presumptive S/G2 cells (DAPI High, 1.5–2.5) after treatment with veliparib (Fig. 3D–F; Fig. S4B). Co-treatment with PUGNAc all but abrogated the veliparib effect. A similar pattern was observed for RAD51 foci persistence at 24 h, where PUGNAc similarly suppressed the veliparib effect (Fig. S6).

Notably, veliparib and PUGNAc appeared to have more modest effects on both the formation (2 h) and persistence (24 h) of 53BP1 foci in S/G2 cells compared to marked impacts on both BRCA1 and RAD51 foci. Among potential mechanisms consistent with these results and prior studies, it is possible that OGT and PARP1 substantially limit DSB repair by HR but their activity might only marginally increase repair by NHEJ.

### O-GlcNAcylation suppresses hyper-resection induced by veliparib upon irradiation

**Fig. 4.**
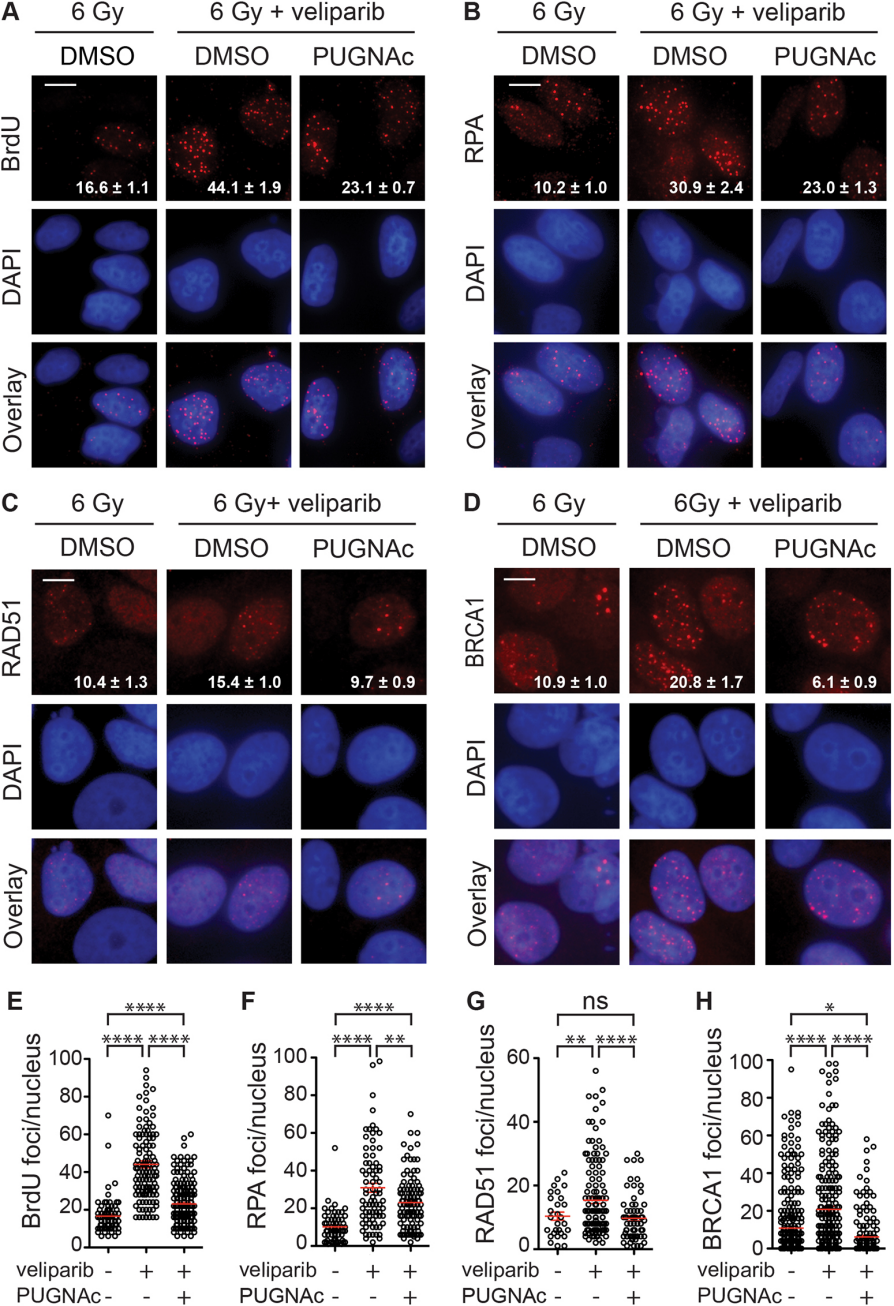
**Increased end resection after irradiation induced by veliparib is suppressed by O-GlcNAcylation.** (A) MCF7 cells were incubated with 10 μM BrdU for 24 h, then treated for 1 h before 6 Gy with DMSO with or without 10 µM veliparib or 50 μM PUGNAc+10 µM veliparib, fixed after 24 h and single strand DNA was detected with anti-BrdU antibody under non-denaturing conditions. Representative images show BrdU foci in red with DAPI in blue. (B–D) Cells were treated for 1 h before 6 Gy with DMSO with or without 10 µM veliparib or 50 μM PUGNAc+10 µM veliparib, fixed after 24 h and probed for RPA (B), RAD51 (C) or BRCA1 (D). (E–H) Plots of BrdU (E), RPA (F), RAD51 (G) and BRCA1 (H) foci per nucleus at 24 h after 6 Gy, showing that PUGNAc reduced the veliparib-induced hyperresection. Scale bars: 20 μm. Values on the images are mean±s.e.m. foci per nucleus. (*n*>75). For plots, red bars indicate mean±s.e.m. (*n*>75). ****P<0.0001; **P<0.01; *P<0.05; ns, *P*>0.05 (two-tailed unpaired *t*-test).

Consistent with other studies implicating PARP1 in suppressing HR repair, Caron et al. (2019) observed that cells treated with PARPi, including veliparib, display markedly increased resection at DSBs. To examine whether PARylation and O-GlcNAcylation might converge on resection, we used exposure of BrdU and formation of RPA foci to detect ssDNA formation after irradiation. For the BrdU assay, culturing MCF7 cells in BrdU for one doubling allows incorporation into newly synthesized chromosomal DNA strands. Upon resection, BrdU is unmasked so that anti-BrdU can bind and detect ssDNA (Mukherjee et al., 2015). The resulting foci of immunoreactivity likely reflect long range resection at individual DSBs. When BrdU-labeled cells were irradiated with 6 Gy and examined at 24 h, cells pretreated with veliparib displayed significantly increased nuclear BrdU foci, consistent with PARPi-induced hyper-resection (Fig. 4A,E). As previously observed (Erdal et al., 2017), conditions that increased BrdU nuclear foci also induced cytoplasmic BrdU foci. Co-treatment with PUGNAc suppressed much of veliparib's effects on BrdU foci persistence. Confirming the BrdU assay, veliparib significantly increased the number of RPA foci at 24 h after 6 Gy, while co-treatment with PUGNAc suppressed this change (Fig. 4B,F). To determine whether the effects of veliparib with or without PUGNAc on DNA end resection after IR extend to other cells, we tested a second HR-proficient cell line, the cervical cancer cell line HeLa. Similar to MCF7 cells, veliparib significantly increased RPA foci in HeLa at 24 h post-IR, while PUGNAc co-treatment attenuated this effect (Fig. S7A–C).

Treating cells with the MRE11 inhibitor mirin prior to irradiation prevented RPA foci formation by 3 h and suppressed the effects of veliparib with or without PUGNAc (Fig. S8). Consistent with the pattern for BrdU and RPA foci, RAD51 foci at 24 h after 6 Gy were also increased in veliparib-treated cells but this effect was almost fully suppressed by co-treatment with PUGNAc (Fig. 4C,G). The pattern shared between BrdU, RPA and RAD51 foci was qualitatively distinct from that for BRCA1 foci (Fig. 4D,H). Notably, BRCA1 is subject to both O-GlcNAcylation (Averbek et al., 2021) and PARylation, with the latter limiting resection (Lodovichi et al., 2023).

**Fig. 5.**
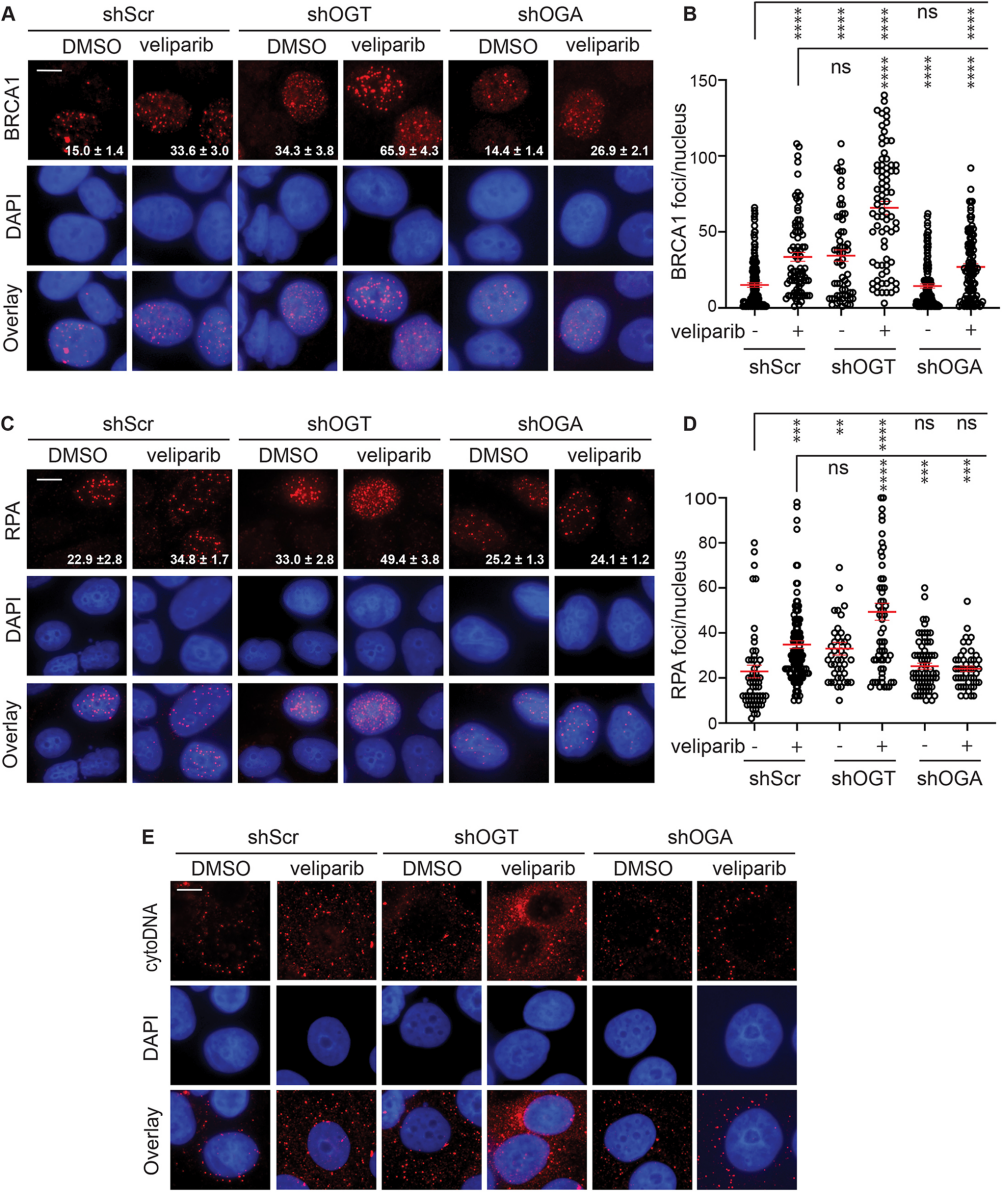
**Targeting OGT or OGA affects RPA and BRCA1 foci persistence induced by veliparib and irradiation.** (A,B) shScr, shOGT and shOGA were induced for 48 h with 1 µg/ml doxycycline and cells were treated with 0 or 10 μM veliparib for 1 h prior to 6 Gy, fixed after 24 h and stained for BRCA1. Shown are representative images (A) and plots of BRCA1 foci per nucleus (B). (C,D) Cells were treated as in A and stained for RPA. Shown are representative images (C) and plots of RPA foci per nucleus (D), showing that shOGT increased RPA foci on its own and further enhanced veliparib-induced hyperresection, whereas shOGA attenuated veliparib-induced hyperresection. (E) shScr, shOGT and shOGA cells were treated as in A and then lightly permeabilized, fixed and then stained with anti-DNA to detect cytoDNA. Shown are representative pseudo-colored images, with cytoDNA in red, DAPI counterstain in blue, and overlays. Consistent with their effects on DNA end resection, shOGT promoted veliparib-induced cytoDNA accumulation, whereas shOGA attenuated it. Scale bars: 20 μm. Values on the images are mean±s.e.m. foci per nucleus. (*n*>75). For plots, red bars indicate mean±s.e.m. (*n*>75). ****P<0.0001; ***P<0.001; **P<0.01; ns, *P*>0.05 (two-tailed unpaired *t*-test).

Given limitations of small-molecule inhibitors, we complemented pharmacological inhibition with analysis of inducible OGT and OGA knockdowns. Using MCF7 cells expressing shScr, shOGT or shOGA, cells were treated with doxycycline for 48 h to induce each shRNAmir, treated with 6 Gy with or without veliparib and then fixed at 24 h and stained for BRCA1 (Fig. 5A,B). shScr cells demonstrated the expected increase in BRCA1 foci following veliparib treatment. shOGT on its own yielded similar BRCA1 foci persistence to shScr plus veliparib while shOGT plus veliparib led to a further increase in persistent BRCA1 foci. Consistent with the effects of PUGNAc shown in Fig. 3D,F, shOGA largely overcame the effects of veliparib on BRCA1 foci persistence.

To examine whether modulating O-GlcNAcylation leads to corresponding changes to resection, each of the shRNAmirs was induced, the cells treated with 6 Gy with or without veliparib, and cells examined for RPA foci at 24 h (Fig. 5C,D). shScr cells displayed persistent RPA foci that were significantly more abundant after veliparib treatment. As with BRCA1, shOGT on its own resulted in persistent RPA foci comparable to that seen for shScr after 6 Gy plus veliparib. Treating the shOGT cells with veliparib led to a further increase in persistent RPA foci, whereas shOGA cells displayed similar RPA foci numbers whether treated with veliparib or not. We also examined RPA foci formation at 2 h (Fig. S9A,B) and observed a similar pattern. Collectively, these findings confirm O-GlcNAcylation as a regulator of resection.

Fragments of both single- and double-stranded DNA formed as a result of processing and repair of chromosomal DSBs can be released from the nucleus, resulting in accumulation of cytosolic DNA (cytoDNA) that is detected by nucleic acid sensors, leading to an inflammatory response (Miller et al., 2021). One consequence of the hyper-resection after irradiation observed upon blocking PARylation and/or O-GlcNAcylation might be increased cytoDNA. Immunostaining using antibodies recognizing both ssDNA and dsDNA in partially permeabilized cells detects foci of immunoreactivity that are consistent with cytoDNA fragments. As expected, shScr cells treated with veliparib and 6 Gy displayed increased cytoDNA over 6 Gy alone (Fig. 5E). Irradiated shOGT cells displayed high cytoDNA staining that was markedly enhanced in veliparib-treated cells. By contrast, veliparib failed to increase cytoDNA in shOGA cells, suggesting a protective role for O-GlcNAcylation in limiting cytoDNA.

### The effects of protein O-GlcNAcylation on DNA end resection are independent of PARP1 but dependent on EZH2

**Fig. 6.**
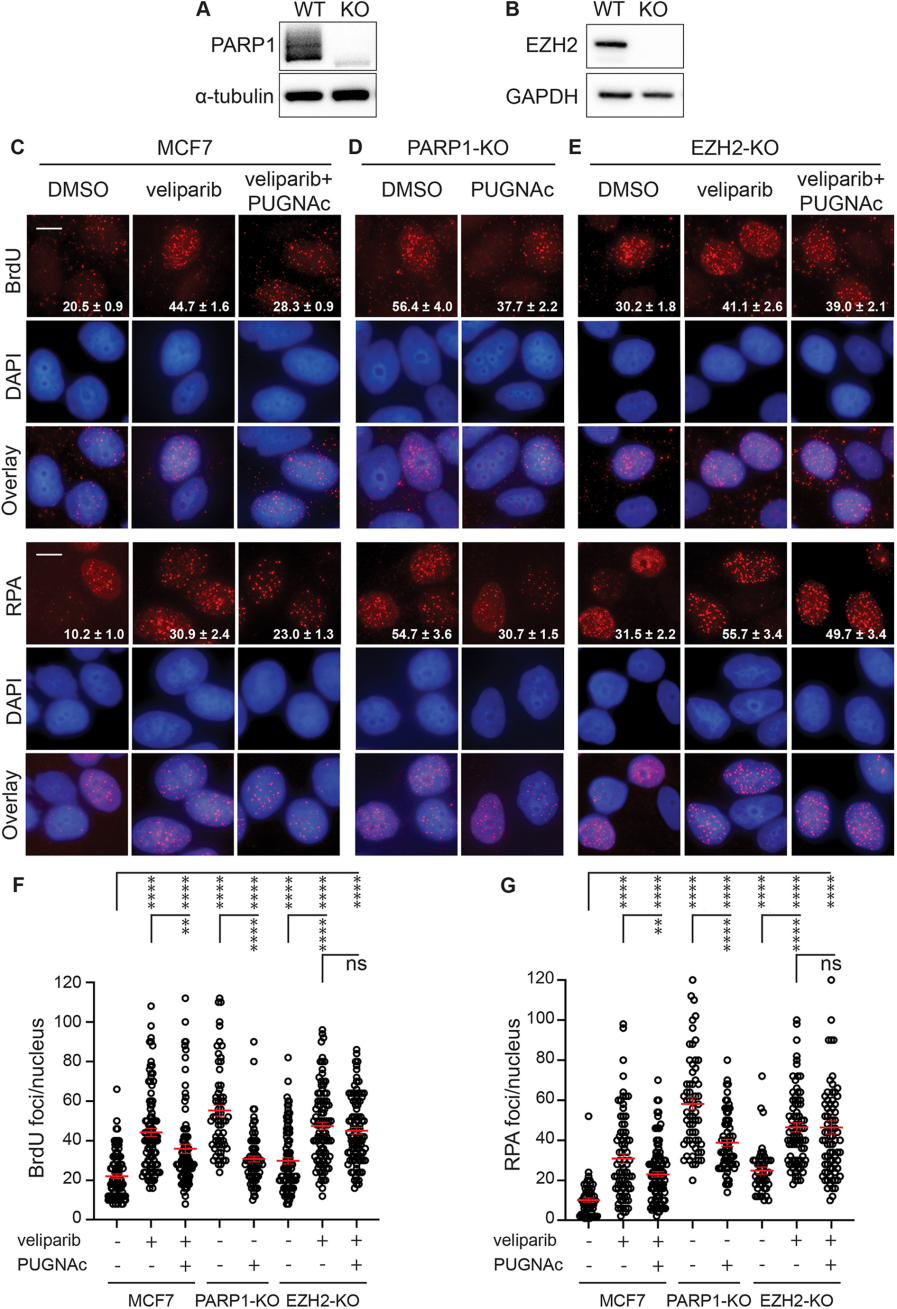
**The effects of O-GlcNAcylation on end resection are independent of PARP1 but dependent on EZH2.** (A,B) Western blot validation of PARP1 (A) and EZH2 (B) knockout (KO) pools. MCF7 cells were electroporated with CRISPR RNP formed with sets of three sgRNAs targeting the PARP1 or EZH2 initiation codons or three non-targeted guides used as wild-type (WT) control. Blot images are from a single western blot performed as an orthogonal validation of knockout, in addition to genotypic validation performed by PCR at the targeted locus. (C–E) MCF7 WT (C), PARP1-KO (D), and EZH2-KO (E) cells were incubated with 10 μM BrdU for 24 h, followed by 1 h treatment before 6 Gy with 0 or 10 µM veliparib with or without 50 μM PUGNAc. After 24 h, cells were fixed and stained with anti-BrdU under non-denaturing conditions (upper) or with anti-RPA (lower). (F,G) Plots of BrdU (F) or RPA (G) foci per nucleus for cells treated as in C–E, showing that PUGNAc reduced hyperresection in PARP1-inhibited or PARP1-KO cells, but not in EZH2-KO cells. Scale bars: 20 μm. Values on the images are mean±s.e.m. foci per nucleus. (*n*>75). For plots, red bars indicate mean±s.e.m. (*n*>75). ****P<0.0001; **P<0.01; ns, *P*>0.05 (two-tailed unpaired *t*-test).

The above studies place OGT-dependent O-GlcNAcylation in parallel with or downstream of PARP1 activity with respect to limiting single-strand resection and resulting effects on DSB repair. However, considering that using veliparib to inactivate PARP1 is limited by confounding effects of PARP1 DNA binding and trapping, we generated a pool of PARP1-KO MCF7 cells using CRISPR RNP electroporation (Fig. 6A). Without IR, PARP1-KO cells displayed an elevated baseline amount of 53BP1 foci compared to that in control MCF7 cells. This increase was mitigated by PUGNAc pretreatment, which enhanced O-GlcNAcylation in PARP1-KO cells to levels comparable to those in control MCF7 cells (Fig. S10A–C). We next examined resection at 24 h after 6 Gy by BrdU assay and RPA staining (Fig. 6C,D,F,G). Compared to control cells, PARP1-KO cells displayed significantly increased DNA end resection in each assay, even beyond that induced by veliparib. Both BrdU and RPA foci persistence in PARP1-KO cells were markedly suppressed by PUGNAc, suggesting that O-GlcNAcylation can mediate its effects independently of PARP1.

To identify potential downstream effectors linking O-GlcNAcylation to DNA end resection, we focused on EZH2, the catalytic subunit of PRC2, which mediates histone H3 K27 trimethylation. In prior work, we implicated the methyltransferase activity of EZH2 in mediating the effects of O-GlcNAcylation on DSB repair (Efimova et al., 2016). MCF7 EZH2-KO cells were generated using CRISPR/Cas9 (Fig. 6B). Without IR, EZH2-KO cells displayed a modest increase in baseline 53BP1 foci compared to the number in control MCF7 cells and this increase was further enhanced by veliparib treatment. However, unlike in PARP1-KO cells, although pretreatment of EZH2-KO cells with PUGNAc raised O-GlcNAcylation to levels comparable to those in control cells, PUGNAc did not reduce the increased 53BP1 foci in unirradiated EZH2-KO cells, either with or without veliparib (Fig. S10A–C). These findings suggest that the influence of O-GlcNAcylation on 53BP1 foci depends on EZH2. We also analyzed DNA end resection at 24 h after 6 Gy using the BrdU and RPA assays in these cells (Fig. 6C,E,F,G). On its own, EZH2-KO cells displayed increased BrdU and RPA foci after irradiation, suggesting that EZH2 might negatively regulate end resection. Veliparib further increased BrdU and RPA foci in EZH2-KO cells, suggesting that PARP1 remains functional in these cells. PUGNAc failed to suppress the effect of veliparib in EZH2-KO cells, suggesting that EZH2 is a mediator of the role of OGT in restraining end resection. To further characterize the resection defect in the PARP1 and EZH2 knockouts, we compared BRCA1 foci persistence at 24 h after 6 Gy in MCF7 cells with or without veliparib to PARP1-KO and EZH2-KO cells. Much like the effect of veliparib treatment, both PARP1-KO and EZH2-KO displayed increased BRCA1 foci (Fig. S11A,B).

Considering our observations linking end resection to cytosolic DNA, we next assessed whether the PARP1 and/or EZH2 knockouts might directly impact cytoDNA levels after irradiation (Fig. S11C). Mirroring their effects on DNA end resection, PARP1-KO and EZH2-KO each displayed increased cytoDNA accumulation at 24 h after 6 Gy compared to MCF7 controls. Veliparib treatment further increased cytoDNA in the EZH2-KO cells. Although PUGNAc fully suppressed cytoDNA levels in PARP1-KO cells, it had no appreciable impact on cytoDNA accumulation in veliparib-treated EZH2-KO cells. These results place EZH2 downstream of O-GlcNAcylation in limiting resection and cytosolic DNA accumulation following PARP inhibition.

## DISCUSSION

This study explores a novel connection between the Warburg effect, resulting activation of the HBP and DNA DSB repair. Our results implicate glycolysis, the HBP and resulting protein O-GlcNAcylation in limiting 5′ to 3′ end resection and defective DSB repair after irradiation of PARPi-treated HR-proficient breast cancer cells. Using genetic and pharmacological perturbations of OGT and OGA in the HR-proficient cell line MCF7, we demonstrate that O-GlcNAcylation influences the accumulation of DNA repair foci, the recruitment of HR factors, and the extent of ssDNA formation following irradiation. These effects occur in parallel with and, in some contexts, independently of PARP1 activity. We further show that EZH2 is required for the suppression of resection by O-GlcNAcylation. Together, these findings suggest that O-GlcNAcylation contributes to the regulation of repair pathway choice by limiting resection in S/G2 cells, and that its inhibition can enhance radiosensitization when combined with PARP inhibition.

### PARP inhibition in HR-proficient cells – repair imbalance and genomic instability

PARPi have shown their greatest clinical utility in tumors lacking BRCA1 or bearing other mutations that confer HRD, yet considerable evidence implicates PARP1 in modulating recombination repair in HR-proficient cells (Chaudhuri and Nussenzweig, 2017). Mechanistically, PARP1 might serve a role in initiating 5′ to 3′ resection, potentially through recruitment of the MRN complex and BRCA1 to DSBs, and thereby promote HR. Other results suggest PARP1 blocks resection to limit HR, leaving considerable uncertainty.

Combining PARP inhibition with irradiation has long been known to delay DSB repair and increase persistent IR-induced γH2AX and 53BP1 foci (IRIF). As previously observed by Caron et al. (2019), we found that PARP inhibition was also associated with increased markers of resection and recombination, including persistent BrdU, RPA and RAD51 foci. This is consistent with shifting repair pathway choice away from NHEJ and toward HR in cells with S/G2 DNA content. We observed that the persistent 53BP1 foci appeared limited to cells that were actively incorporating EdU at the time of irradiation, indicating that the repair defects were linked to DSBs formed during DNA replication. Although increased resection might accelerate DSB repair in some circumstances, here it was associated with persistent ssDNA, suggesting insufficient HR activity to meet demand. Furthermore, the pattern of increased BrdU, RPA and RAD51 foci, unresolved damage and cytoDNA is consistent with a model in which resection far exceeds any benefits and becomes toxic.

Our work adds to published results that associate PARP inhibition with deregulated end resection, detected by persistence of ssDNA and RPA foci, and elevated recombination activity, evidenced by elevated RAD51 foci and increased sister chromatid exchanges. Although seemingly inconsistent, this pattern suggests increased resection is sufficient not only to enhance HR but also to overwhelm its capacity. In its normal role, PARP1 might support DSB repair by limiting end resection and thereby metering the formation of substrates for HR repair. Furthermore, our findings support the idea that radiosensitization due to PARP inhibitors might result not from blocked DNA repair per se but from excessive ssDNA formation that outpaces the capacity of the cell to resolve it. Hyper-resection may drive repair defects, checkpoint activation and inflammatory signaling, each of which could enhance radiation sensitivity.

### Cancer metabolic reprogramming as a regulator of repair pathway choice via O-GlcNAcylation-mediated suppression of end resection

Metabolic rewiring in cancer, including the Warburg effect, resulting in enhanced glycolytic flux and increased activity of the HBP, leads to elevated protein O-GlcNAcylation. OGT, the enzyme responsible for this modification, has been implicated in regulation of the DNA damage response, including DSB repair (Efimova et al., 2019; Wang et al., 2016). Prior studies have shown that O-GlcNAcylation can affect both repair kinetics and pathway usage, but the specific mechanisms remain incompletely resolved. OGT targets multiple chromatin-associated and DNA repair proteins, including the MRN complex, BRCA1 and chromatin regulators, implying the potential for widespread influence on repair processes. Recent reviews highlight the diversity and complexity of O-GlcNAcylation effects on genome maintenance, suggesting context-dependent or even opposing roles depending on the timing and targets of modification (Zhu et al., 2024).

In this study, we examined O-GlcNAcylation in irradiated cells treated with veliparib and found that its loss phenocopied key features of PARP1 inhibition – increased RPA and BrdU foci, prolonged DNA repair signaling, increased RAD51 loading and cytoDNA accumulation. These phenotypes were enhanced when OGT knockdown was combined with PARPi, whereas OGA knockdown or inhibition with PUGNAc limited resection, whether PARP1 was active or not. These results suggest that O-GlcNAcylation limits resection through a mechanism that acts in parallel to PARP1 enzymatic activity.

Although the precise mechanism remains to be defined, our findings support the concept that O-GlcNAcylation serves as a metabolic checkpoint on DSB processing. Whether this is mediated by chromatin modification, regulation of resection machinery or transcriptional effects remains unclear. Nonetheless, the ability of a nutrient-sensitive post-translational modification to modulate resection and thereby limit substrates for HR points to an underappreciated layer of metabolic control over repair pathway choice.

### EZH2 functions downstream of OGT in limiting end resection at DSBs

We revisited the role of EZH2 in DSB repair based on prior studies identifying it as a functional target of OGT (Efimova et al., 2016; Lo et al., 2018). The canonical role of EZH2 is as a subunit of PRC2, serving as the catalytic subunit that trimethylates histone H3 K27 (H3K27me3), a mark associated with gene repression. However, it is unclear whether its DNA repair function(s) require protein methylation, let alone regulation of gene expression, chromatin architecture or transcription factor recruitment. EZH2 has PRC2-independent functions and can modify non-histone substrates including methylating PARP1 as part of a reciprocal inhibition relationship (Caruso et al., 2018; Meng et al., 2024; Yamaguchi et al., 2018). Notably, prior studies have documented recruitment of the PRC2 complex and deposition of H3K27me3 marks at DSBs and that this EZH2-dependent modification promotes DSB repair, foci resolution and radio-resistance (Campbell et al., 2013; Chou et al., 2010; Lutze et al., 2021 preprint). Although stable H3K27me3 domains are associated with gene silencing and chromatin condensation, the function of transient K27 trimethylation at DSBs might be distinct. By binding and/or depositing its repressive epigenetic mark, EZH2 may favor NHEJ by pausing RNA Pol II and/or excluding resection proteins. In *Drosophila*, loss of the H3K27 demethylase UTX leads to a shift toward NHEJ in DSB repair in facultative heterochromatin, suggesting that H3K27me3 inhibits HR (Wensveen et al., 2024).

In our experiments, EZH2-KO phenocopied OGT loss, increasing end resection and RPA and RAD51 foci persistence following IR. Notably, OGA inhibition failed to suppress hyper-resection in EZH2-deficient cells, suggesting that EZH2 is required for O-GlcNAcylation to restrain HR. These results may be cell line-dependent as prior studies have implicated EZH2 in protecting against end resection in CARM1-high ovarian cancer cells via enhancing expression of the shieldin subunit MAD2L2/REV7 (Karakashev et al., 2020) or promoting NHEJ in MYC-high medulloblastoma via upregulation of NUPR1 (Yu et al., 2025). A favorable interpretation of our results might ascribe the negative influence of OGT and O-GlcNAcylation on end resection to facilitating formation of H3K27me3 at DSBs by EZH2. Whether depositing this “silencing” mark might reduce chromatin remodeling, exclude RNA Pol II, alter accessibility of resection machinery, and/or otherwise mediate epigenetic control over DSB processing remains to be determined.

Finally, we observed a compound effect on BrdU and RPA foci persistence when EZH2 KO cells were treated with veliparib, suggesting independent activities of EZH2 and PARP1 in limiting resection initiation and/or termination. There is continuing interest in the potential of combining PARPi and EZH2 inhibitors in both HR-deficient and -proficient cancers (Meng et al., 2024; Zhang et al., 2022). These results point to an unanticipated mechanism of synergy linked to deregulated 5′ to 3′ end resection, leading to cellular stress.

A striking observation was that increased resection, irrespective of its origin, appeared to be linked to a proportional increase in cytosolic DNA, suggesting hyper-resection might drive cGAS/STING activation and inflammatory signaling as a consequence. PARPi (Shen et al., 2019) and EZH2 inhibitors (Morel et al., 2021) have each been linked to STING activation and resulting sensitization to checkpoint blockade. Inflammatory signaling may be further amplified upon dual PARP1 and EZH2 inhibition (Yang et al., 2021).

### Cell cycle-dependent resection stress and selective toxicity to proliferating cells

Each of the impacts on radiation response we observed upon loss of PARylation and/or O-GlcNAcylation, including persistent repair foci, increased resection markers, and cytosolic DNA accumulation, was limited to cells in S and/or G2. S/G2 is characterized by the potential for DSB repair by HR, linked to the availability and activation of BRCA1 and other determinants of nucleoprotein filament formation along with access to sister chromatids as repair templates. This cell cycle specificity suggests a strategy for selectively targeting proliferating cells. While S-phase cells are classically viewed as relatively radioresistant due to efficient HR, our results imply that this robustness can be undermined when resection proceeds inappropriately or cannot be terminated. The consequence may not be immediate cytotoxicity, but rather persistent intermediates, checkpoint activation, and ultimately outcomes like mitotic failure or inflammatory signaling.

Importantly, the observed sensitivity may not arise from direct inhibition of DNA replication or repair but rather from deregulation of the pathways that control DNA damage resolution. Interventions that skew DSB repair pathway choice toward HR to the point of overwhelming its capacity may expose proliferating tumor cells to resection stress, while sparing non-cycling populations.

### New targets and mechanisms for radiosensitization

Our findings support a model in which loss of regulatory control over end resection can sensitize HR-proficient tumor cells to IR. PARP1 and OGT inhibition each increased resection markers and delayed the resolution of repair foci, whereas their combination amplified these effects, producing persistent RAD51 and RPA foci and cytosolic DNA accumulation. These phenotypes were enriched in S/G2 cells, where the capacity for HR is normally beneficial but may become deleterious when resection does not lead to repair. In short, inducing DSBs in HR-proficient cells can create a dependency where PARylation and/or O-GlcNAcylation might be required to protect against toxic repair.

Taken together, our findings point to a new strategy to enhance sensitivity to radiation in HR-proficient cancers. Along with supporting cell proliferation and survival, the Warburg effect could help protect tumors from toxic DNA repair via aerobic glycolysis feeding the hexosamine biosynthetic pathway, leading to increased O-GlcNAcylation and a shift away from resection and HR. Whereas overcoming cancer metabolic reprogramming has proved challenging, targeting the downstream mediator EZH2, potentially along with inhibiting PARP1, is quite feasible. Thereby, it might be possible to expose cell proliferation and HR proficiency as an Achilles heel that can be exploited for radiosensitization and potentiation of anti-tumor response. For example, future studies could explore the therapeutic potential of combining PARP inhibitors with reduced O-GlcNAcylation, or, perhaps more simply, with EZH2 inhibition, as a strategy to enhance the radiation response in HR-proficient tumors.

## MATERIALS AND METHODS

### Cell lines and tissue culture

These studies used MCF7 (ATCC), an ER+ breast cancer cell line derived from a metastatic lesion. MCF7 expresses BRCA1 and is HR proficient. For shRNA knockdown studies, we used the MCF7 Tet-On Advanced cell line (Clontech-Takara Bio) which was cultured in high-glucose DMEM supplemented with 10% Tet System-approved fetal bovine serum (Clontech-Takara Bio) and 1% penicillin-streptomycin (Life Technologies) (complete DMEM). The generation and characterization of MCF7 cell lines expressing gene-specific shERWOOD-UltramiR inducible shRNA targeting OGT or OGA (MGEA5), along with a non-targeting scrambled control, all within the pZIP-TRE3GS vector, were as previously described (Efimova et al., 2019). Following induction with 1 μg/ml doxycycline (Sigma-Aldrich) for 48 h, the majority of the cells expressed the ZsGreen fluorescent protein reporter, indicating successful shRNAmiR expression. The effects of shOGT and shOGA on protein O-GlcNAcylation were validated by western blot analysis. mCherry–hCdt1 and mVenus–hGeminin FUCCI reporters in pCSII-EF-MCS, a kind gift of Dr Atsushi Miyawaki at the Riken Brain Sciences Institute, Japan, were introduced into MCF7 cells to track cell cycle progression as described (Sakaue-Sawano et al., 2008). HeLa cells were obtained from ATCC and cultured in complete DMEM. All cell lines were tested for mycoplasma contamination and authenticated by short tandem repeat profiling (IDEXX BioResearch) prior to experimentation. Experiments were conducted within 3 to 10 passages after cell line development.

### Generation of knockout pools by CRISPR RNP electroporation

To generate MCF7 cell lines deficient in PARP1 or EZH2, sets of three pre-designed single-guide RNAs (sgRNAs) targeting human PARP1 or EZH2, along with recombinant SpCas9 nuclease, were purchased from Synthego. Sets of sgRNA targeting sequences used for PARP1 were 5′-AAGAAGACAGCGGAAGCUGG-3′, GUGGCCCACCUUCCAGAAGC-3′, 5′-UUCUAGUCGCCCAUGUUUGA-3′ and for EZH2, 5′-CUCUGAACAGAUUUAGCAUU-3′, 5′-UGCGCCUGGGAGCUGCUGUU-3′, 5′-AAUAAUUGCACUUACGAUGU-3′. For transfection, sets of sgRNAs and SpCas9 were mixed in a 9:1 molar ratio and incubated at room temperature for 10 min to form ribonucleoprotein (RNP) complexes. RNPs (7 μl) were then mixed with 5×10^4^ MCF7 cells in 5 μl of Buffer R (Thermo Fisher Scientific) and transfected using the Neon Electroporation System (Thermo Fisher Scientific) with two pulses at 1250 V, 20 ms pulse width. After transfection, cells were immediately transferred to pre-warmed culture medium to recover in culture and form knockout pools.

Pools of proliferating cells were examined to confirm targeting by PCR amplification of the targeted genomic regions, followed by Sanger sequencing and querying the Synthego Inference of CRISPR Edits (ICE) Tool (Conant et al., 2022). Genomic DNA was extracted using QuickExtract DNA Extraction Solution (Lucigen) according to the manufacturer's instructions. PCR amplification was performed using AmpliTaq Gold 360 Master Mix (Thermo Fisher Scientific). Primers for PARP1 were forward primer: 5′-GATGCTGAGTCCAGGAGGTG-3′, reverse primer: 5′-GGCTGTCCTCCTTTCACAGA-3′ and sequencing primer: 5′-GGCTGTCCTCCTTTCACAGAT-3′ and for EZH2, forward primer: 5′-AGCTAATAGTGTGATCTACAGCAGT-3′, reverse primer: 5′-ACCACTTGCAATAAAATAACAGATGTG-3′, and sequencing primer: 5′-AATAGTGTGATCTACAGCAGTCATTAACAG-3′. A 100% editing efficiency was observed for each pool.

### Western blotting

The effects of shOGT and shOGA on OGT and OGA expression and on protein O-GlcNAcylation were validated by western blot analysis. MCF7 cells expressing shScr, shOGT or shOGA were treated with doxycycline for 48 h to induce each shRNAmir expression. Knockdown efficiency was assessed by western blotting using anti-OGT (1:1000, Abcam; ab177941) and anti-OGA (1:1000, Cell Signaling Technology; E9C5U) antibodies. Global O-GlcNAcylation levels were evaluated using an anti-O-GlcNAc antibody (1:1000, Abcam; ab2739). As a positive control for increased O-GlcNAcylation, shScr cells were treated with 50 µM PUGNAc for 24 h.

O-GlcNAcylation was examined under non-irradiated (NIR) conditions, at indicated time points following 6 Gy IR, and 24 h after IR or 10 µM veliparib treatment. Additional conditions included treatment for 24 h with 50 µM PUGNAc (OGA inhibitor), 25 µM OSMI-1, or 2.5 µM ST060266 (OGT inhibitors). Please see the sources of the small molecules below. Where indicated, the same membrane was sequentially probed with anti-OGT, stripped using Restore PLUS Western Blot Stripping Buffer (Thermo Fisher Scientific; 46430), reprobed with anti-O-GlcNAcylation antibody, stripped again, and finally probed with anti-β-actin (1:5000; Proteintech, HRP-66009).

To assess knockout efficiency, PARP1 or EZH2 protein expression in the knockout pools was evaluated by western blot analysis using an anti-PARP1 antibody (1:1000, Cell Signaling Technology; 9532T) or anti-EZH2 antibody (1:1000, Abcam; ab191080). The effect of PUGNAc on O-GlcNAcylation was also assessed in PARP1-KO and EZH2-KO cells. Whole-cell lysates were prepared using RIPA lysis buffer (Thermo Fisher Scientific) supplemented with protease and phosphatase inhibitors (Thermo Fisher Scientific). Protein samples were separated on NuPAGE 4-12% Bis-Tris precast gels (Invitrogen) and transferred onto nitrocellulose membranes (Millipore). Membranes were blocked in 5% non-fat milk in TBST, then incubated with primary antibodies. Anti-GAPDH (1:5000, Proteintech, HRP-60004), -β-actin (1:5000, Proteintech, HRP-66009) or -α-tubulin (1:5000, Proteintech, HRP-66031) antibodies were used as loading controls. Detection was performed using HRP-conjugated secondary antibodies (Thermo Fisher Scientific; NA934VS or NA931) and enhanced chemiluminescence (ECL) substrate (Thermo Fisher Scientific), and signals were imaged with an iBright Imaging System (Thermo Fisher Scientific).

### Single-cell electrophoresis comet assay

Neutral comet assays were performed on MCF7 cells stably transfected with inducible shRNAmiRs, seeded at 10^5^ cells per well in six-well plates, and induced with 1 μg/ml doxycycline for 48 h prior to irradiation. At 24 h after exposure to 6 Gy, cells were mixed with Comet LM Agarose and subjected to single-cell electrophoresis on Comet Slides (Trevigen) following the manufacturer's protocol. Slides were imaged using a Zeiss Axiovert 40 microscope equipped with a 20× Plan-Neofluar objective and an AxioCam camera. Comet moment and comet tail percentage were analyzed using an ImageJ comet assay macro.

### Immunofluorescence imaging of DNA repair foci

For imaging γH2AX and 53BP1 foci, MCF7 cells with inducible shRNA were seeded on coverslips at 2.5×10^4^ cells per well in 24-well plates and induced with 1 μg/ml doxycycline for 48 h. Cells were treated with veliparib 1 h prior to 6 Gy. At 24 h post-irradiation, cells were fixed with 4% paraformaldehyde (PFA) in PBS and permeabilized with 0.5% Triton X-100 for 10 min. Non-irradiated control cells were fixed in parallel. Following blocking with 5% BSA, cells were incubated overnight at 4°C with primary antibodies against γH2AX (Millipore; 05-636; 1:1000) or 53BP1 (Novus Biologicals; NB100-304; 1:1000). After washing with PBS, fluorescent secondary antibodies (Jackson ImmunoResearch) were applied for 1 h at room temperature. Nuclei were stained with DAPI at a final concentration of 1 μg/ml, and coverslips were mounted using ProLong Gold antifade reagent (Invitrogen).

For imaging 53BP1, BRCA1 and RAD51 foci, MCF7 cells were seeded and treated as previously described. Cells were fixed at either 2 or 24 h post-irradiation, as indicated. The primary antibodies used were against 53BP1, BRCA1 (1:1000, Santa Cruz Biotechnology; sc-6954) and RAD51 (1:1000, Novus Biologicals; NB100-148). Secondary antibody staining, DNA counterstaining with DAPI and slide mounting were performed as described above. After mounting and drying, images were captured using a Zeiss Axiovert 40 CFL microscope with a 40× Plan-Neofluar objective and an AxioCam digital camera controlled by AxioVision 4.8 software. Following imaging, the number of foci per nucleus in multiple microscopic fields was determined by manual counting, and data are presented as mean±s.e.m. Images were pseudo-colored using ImageJ or Adobe Photoshop.

For small-molecule compound treatment, veliparib was obtained from AbbVie; PUGNAc from Toronto Research Chemicals; OSMI1 and ST060266 from Selleck Chemicals; mirin and N-GlcNAc from Sigma-Aldrich.

### DNA end resection assays

MCF7 cells were seeded on coverslips at 2.5×10^4^ cells per well in 24-well plates and incubated with BrdU at a final concentration of 10 μg/ml for 24 h. Cells were then treated with the indicated inhibitors alone or in combination 1 h prior to irradiation. At 2 and 24 h post-irradiation, cells were washed with PBS and incubated with extraction buffer (10 mM PIPES pH 7.0, 100 mM NaCl, 300 mM sucrose, 3 mM MgCl_2_, 1 mM EGTA and 0.5% Triton X-100) for 10 min on ice. Cells were then fixed with 4% PFA in PBS for 20 min on ice and permeabilized with 0.5% Triton X-100 for 10 min. After blocking with 5% BSA, cells were incubated overnight at 4°C with a primary antibody against BrdU (1:100, BD Biosciences; 347580). For RPA immunostaining, the same protocol was followed for MCF7 or HeLa cells, except that BrdU incorporation was omitted, and an anti-RPA antibody was used (1:1000, Abcam; ab2175). Secondary antibody incubation and nuclear staining were performed as described above. Using these methods, under conditions where high levels of BrdU foci are observed in the nucleus, BrdU foci can also appear in the cytoplasm, as previously described (Erdal et al., 2017).

All images were captured using a Zeiss Axiovert 40 CFL microscope with a 40× Plan-Neofluar objective and an AxioCam digital camera controlled by AxioVision 4.8 software. Experiments were performed in biological duplicates or more. After imaging, the number of foci per nucleus in multiple microscopic fields was determined by manual counting, and data are presented as means±s.e.m. Images were pseudo-colored using ImageJ or Adobe Photoshop.

### Detection of cytoDNA

Cells were treated as indicated, fixed with 4% PFA in PBS for 10 min and permeabilized with 0.1% Tween 20 and 0.01% Triton X-100 for 7-10 min. This step is crucial, as it permits antibody penetration across the plasma membrane while preserving nuclear membrane integrity. After blocking with 1% BSA, cells were incubated overnight at 4°C with an anti-DNA antibody (ab27156, 1:1000). Notably, this antibody displays strong reactivity to both ssDNA and dsDNA. Secondary antibody incubation and nuclear staining were performed as described (Spada et al., 2019). All images were captured using a Zeiss Axiovert 40 CFL microscope with a 40× Plan-Neofluar objective and an AxioCam digital camera controlled by AxioVision 4.8 software. Experiments were performed in biological duplicates or more. Images were pseudo-colored using ImageJ or Adobe Photoshop.

### Statistical analysis

All experiments were performed using at least three independent biological replicates unless otherwise noted. For DNA damage foci quantification and comet assay analyses, a minimum of 70 nuclei per condition (typically >100) were evaluated, unless otherwise indicated. Statistical significance for foci counting and comet assays was determined using a two-tailed unpaired *t*-test with Welch's correction, performed using GraphPad Prism 6 software. *P*≤0.05 was considered statistically significant.

## Supplementary Material

10.1242/joces.264322_sup1 Supplementary information
