# Supplementary material for: Cancer metabolism in radiation sensitization – complementary roles of O-GlcNAc transferase and PARP1
Source: J Cell Sci. 2026 Mar 30;139(6):jcs264322. doi: 10.1242/jcs.264322 (PMC13036754; doi:10.1242/jcs.264322)
Supplement: Supplementary information [file joces-139-264322-s1.pdf]

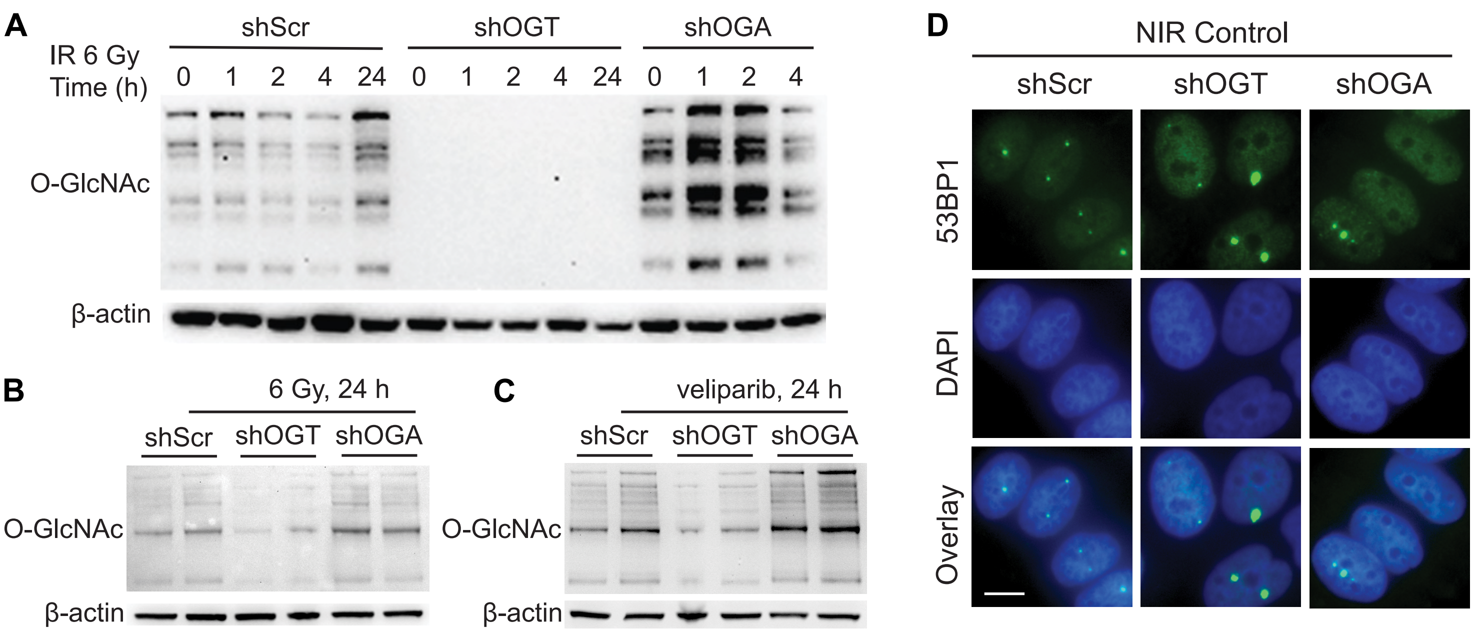

Fig. S1. OGT and OGA knockdown modulate global O-GlcNAcylation without affecting baseline DNA damage foci.

A. Western blot analysis showing the effects of OGT (shOGT) and OGA (shOGA) knockdown on global O-GlcNAcylation before and after 6 Gy. Cells expressing shScr, shOGT, or shOGA were treated with 1 µg/ml doxycycline for 48 h, irradiated with 6 Gy (IR), and harvested at the indicated time points. Non-irradiated (NIR) cells were collected at 0 h. **B-C**. Western blots showing O-GlcNAcylation levels following OGT or OGA knockdown in the presence or absence of 10 µM veliparib. shRNA expression was induced with 1 µg/ml doxycycline for 48 h. Then cells were harvested 24 h after 6 Gy or 10 µM veliparib. Non-irradiated or untreated shScr cells served as controls. For A-C, total cell lysates were analyzed, and β-actin was probed as a loading control. **D**. Representative immunofluorescence images showing 53BP1 foci (pseudo-colored green), DAPI stained DNA (blue), and their overlays in shScr, shOGT, and shOGA cells under non-irradiated (NIR) conditions. Scale bar = 20 µm.

Journal of Cell Science • Supplementary information

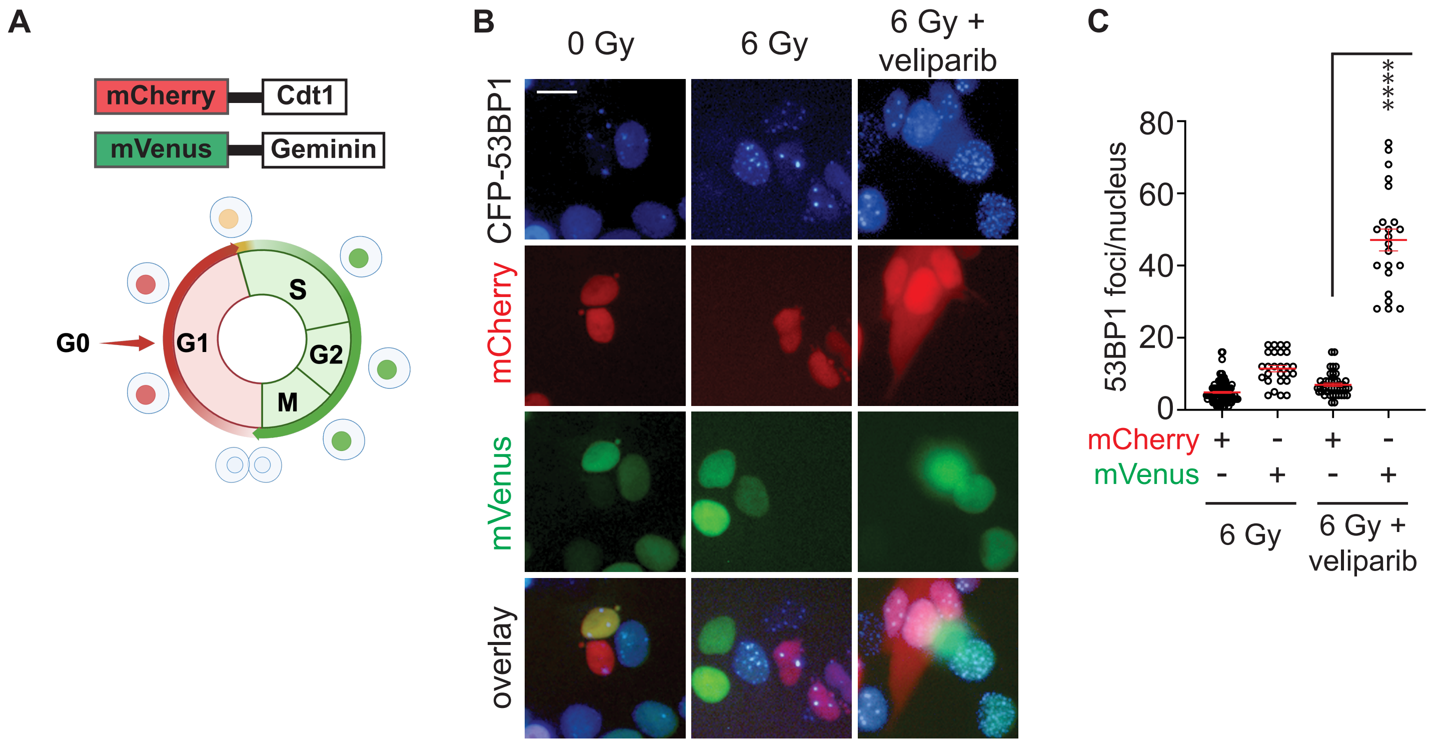

Fig. S2. Veliparib induces foci persistence primarily in S/G2 cells after irradiation. **A.** Schematic illustration of the FUCCI cell cycle reporter system. The G1 phase is characterized by increasing expression of mCherry-Cdt1 (red), the S/G2/M phase by mVenus-Geminin (green), and the G1/S transition by transient co-expression (yellow). **B**. MCF7 cells stably expressing FUCCI cell cycle reporters and 53BP1-CFP were treated with DMSO or 10 µM veliparib for 1 h prior to 6 Gy. After 24 h, cells were imaged by fluorescence microscopy to analyze 53BP1 foci (blue) and FUCCI reporters (red, green). Scale bar = 40 µm. **C**. 53BP1 foci in irradiated cells were quantified per nucleus and grouped by expression of FUCCI reporters, demonstrating that persistent 53BP1 foci were restricted to cells expressing mVenus-Geminin, suggesting a prolonged S/G2 phase associated with persistent DSBs. Red bars indicate mean ± SEM; n > 20. ****, P < 0.0001 (unpaired t-test).

Journal of Cell Science • Supplementary information

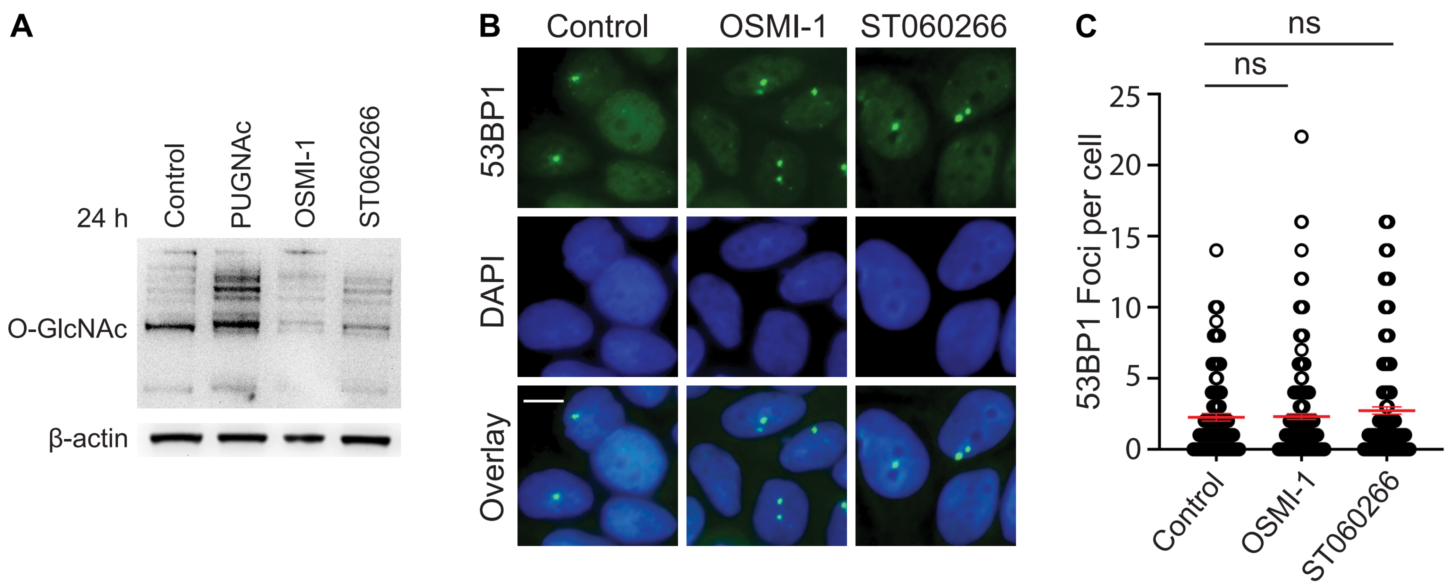

Fig. S3. OGT inhibition reduces O-GlcNAcylation without affecting 53BP1 foci under non-irradiated conditions.

A. Western blot showing O-GlcNAcylation levels after 24 h treatment with the OGA inhibitor PUGNAc (50 µM) or OGT inhibitors OSMI-1 (25 µM) or ST060266 (2.5 µM). Total cell lysates were analyzed, and β-actin was probed as a loading control. **B**. Representative immunofluorescence images showing 53BP1 foci (green), DAPI stained DNA (blue), and their overlays in MCF7 cells treated with vehicle control, OSMI-1 or ST060266 for 24 h. Scale bar = 20 µm. **C**. Quantification of 53BP1 foci per nucleus in individual cells from **B**, showing no effect of OGT inhibitors without irradiation. Red bars indicate mean ± SEM; n > 70. ns, P > 0.05 (unpaired t-test).

Journal of Cell Science • Supplementary information

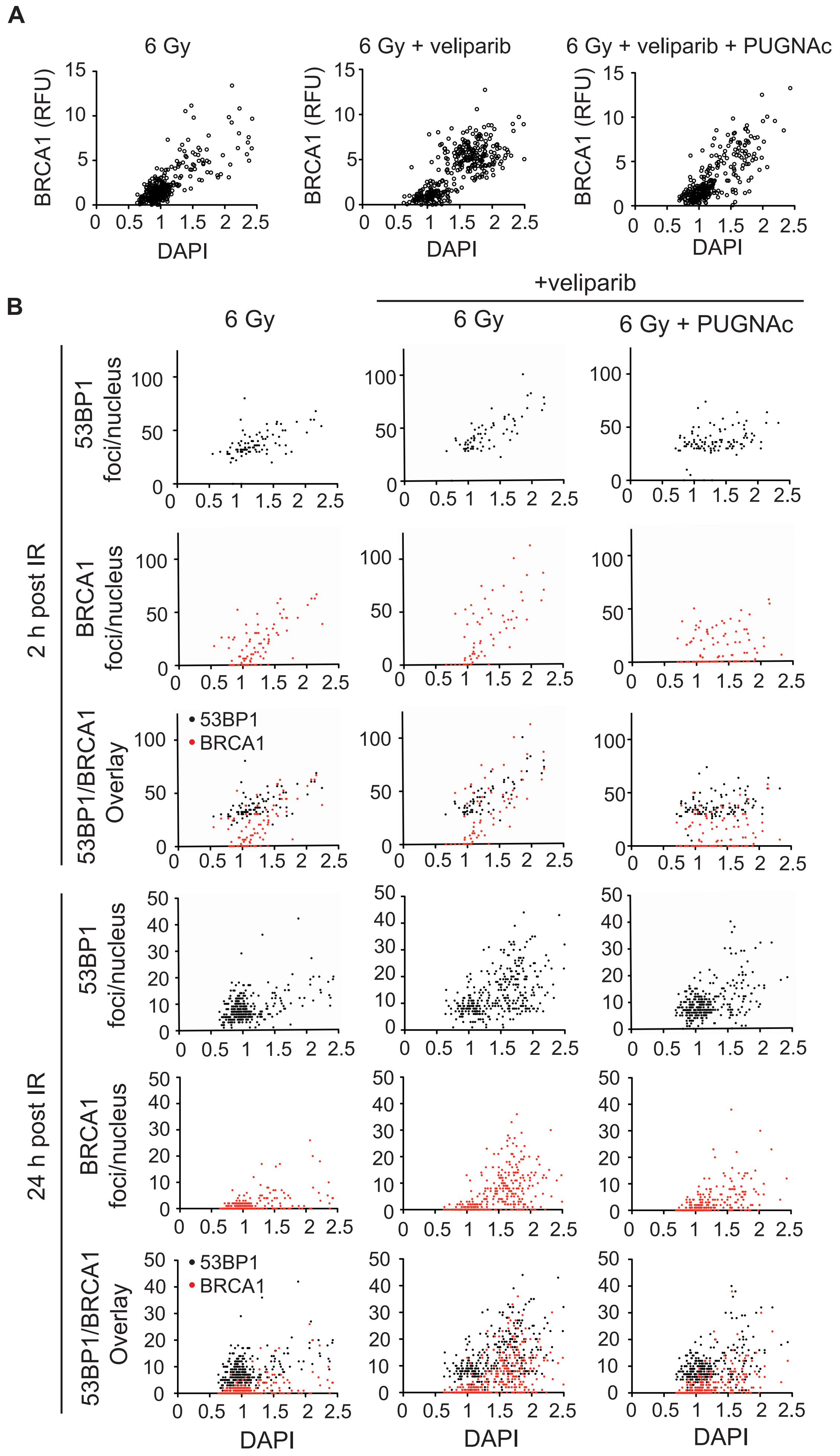

A
6 Gy
6 Gy + veliparib
6 Gy + veliparib + PUGNAc
BRCA1 (RFU)
DAPI
B
+veliparib
6 Gy
6 Gy
6 Gy + PUGNAc
2 h post IR
24 h post IR
53BP1 foci/nucleus
BRCA1 foci/nucleus
53BP1/BRCA1 Overlay
• 53BP1
• BRCA1
DAPI

Fig. S4. O-GlcNAcylation antagonizes veliparib-induced recruitment and retention of BRCA1 after irradiation.

A. MCF7 cells were treated for 1 h prior to 6 Gy with 0 or 10 µM veliparib and DMSO or 50 µM PUGNAc, then fixed after 24 h and stained for BRCA1. To examine whether the expression of BRCA1 was altered in a way that affected the detection of BRCA1 foci, total BRCA1 fluorescence intensity was quantified as relative fluorescence units (RFU) for individual nuclei and plotted vs. normalized DAPI intensity, indicating a similar expression pattern with respect to cell cycle stage. **B**. MCF7 cells were treated for 1 h prior to 6 Gy with 0 or 10 µM veliparib and DMSO or 50 µM PUGNAc, then fixed after 2 h or 24 h and stained for 53BP1 and BRCA1. Shown are plots of 53BP1 (black) and BRCA1 (red) foci per nucleus vs. normalized DAPI intensity. The plots in Fig. S4 were generated from the same imaging data used in Fig. 3. Consistently, veliparib increased the number of 53BP1 and BRCA1 foci more strongly in S/G2 cells (DAPI High, 1.5-2.5) than in G1 cells (DAPI Low, 0.5-1.5).

Journal of Cell Science • Supplementary information

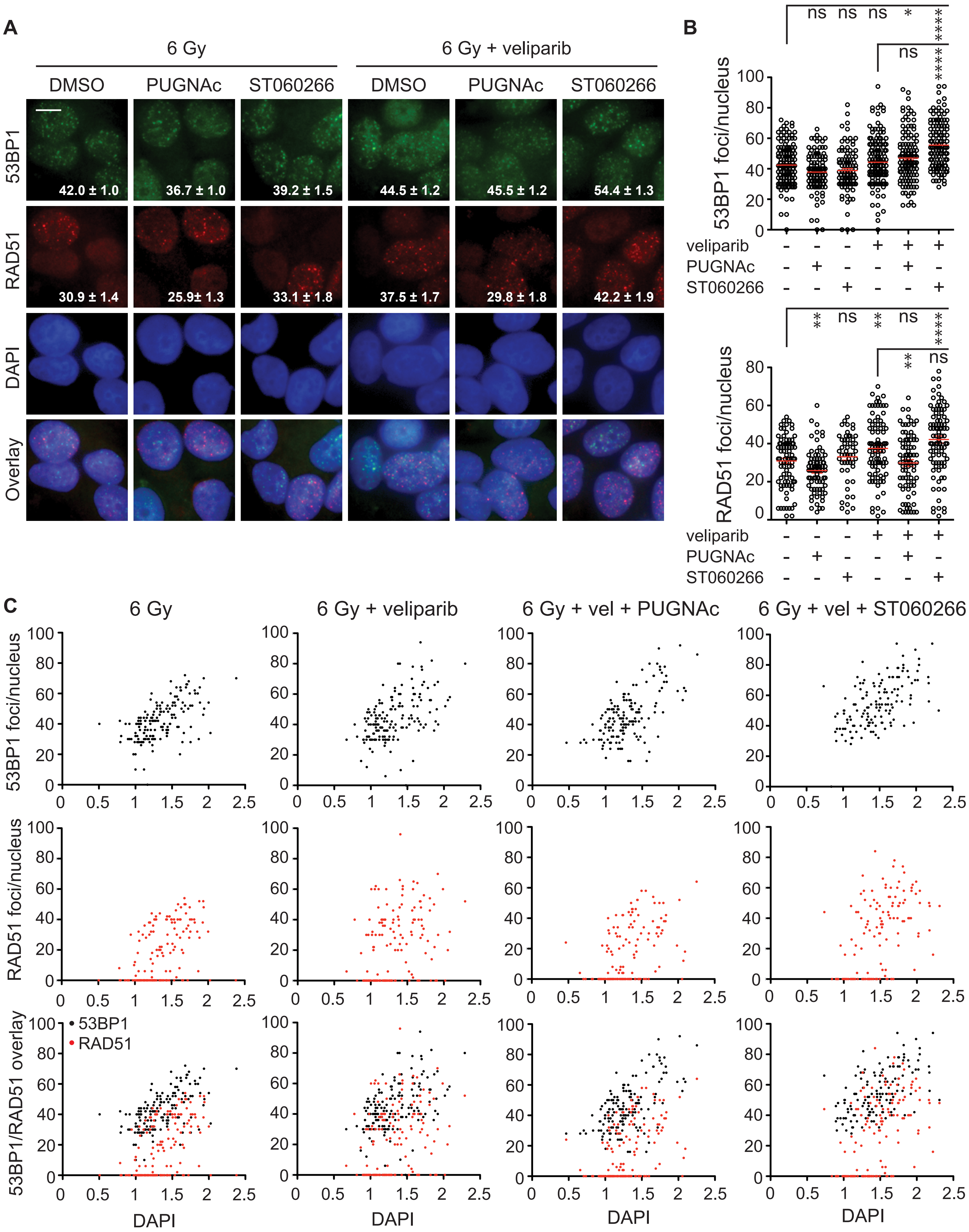
A
6 Gy
6 Gy + veliparib
DMSO
PUGNAc
ST060266
53BP1
RAD51
DAPI
Overlay
42.0 ± 1.0
36.7 ± 1.0
39.2 ± 1.5
44.5 ± 1.2
45.5 ± 1.2
54.4 ± 1.3
30.9 ± 1.4
25.9± 1.3
33.1 ± 1.8
37.5 ± 1.7
29.8 ± 1.8
42.2 ± 1.9
B
53BP1 foci/nucleus
RAD51 foci/nucleus
veliparib
PUGNAc
ST060266
ns
*
****
**
C
6 Gy + vel + PUGNAc
6 Gy + vel + ST060266
53BP1/RAD51 overlay
53BP1
RAD51
DAPI

Fig. S5. OGT and OGA inhibitors differentially affect veliparib-induced RAD51 foci formation after irradiation.

A. Representative images of MCF7 cells treated with DMSO, 50 µM PUGNAc or 2.5 µM OGT inhibitor ST060266 ± 10 µM veliparib for 1 h prior to 6 Gy, then fixed after 2 h and stained for 53BP1 (green) and RAD51 (red) along with DAPI (blue). Scale bar = 20 µm. **B**. Quantification of 53BP1 and RAD51 foci per nucleus. Red bars indicate mean ± SEM; n > 75. ****, P < 0.0001; ***, P < 0.001; **, P < 0.01; *, P < 0.05; ns, P > 0.05 (unpaired t-test). **C**. Shown are plots of 53BP1 (black) and RAD51 (red) foci per nucleus vs. normalized DAPI intensity for individual cells, showing that PUGNAc reduced, whereas ST060266 enhanced, veliparib-induced 53BP1 and RAD51 foci in S/G2-phase cells.

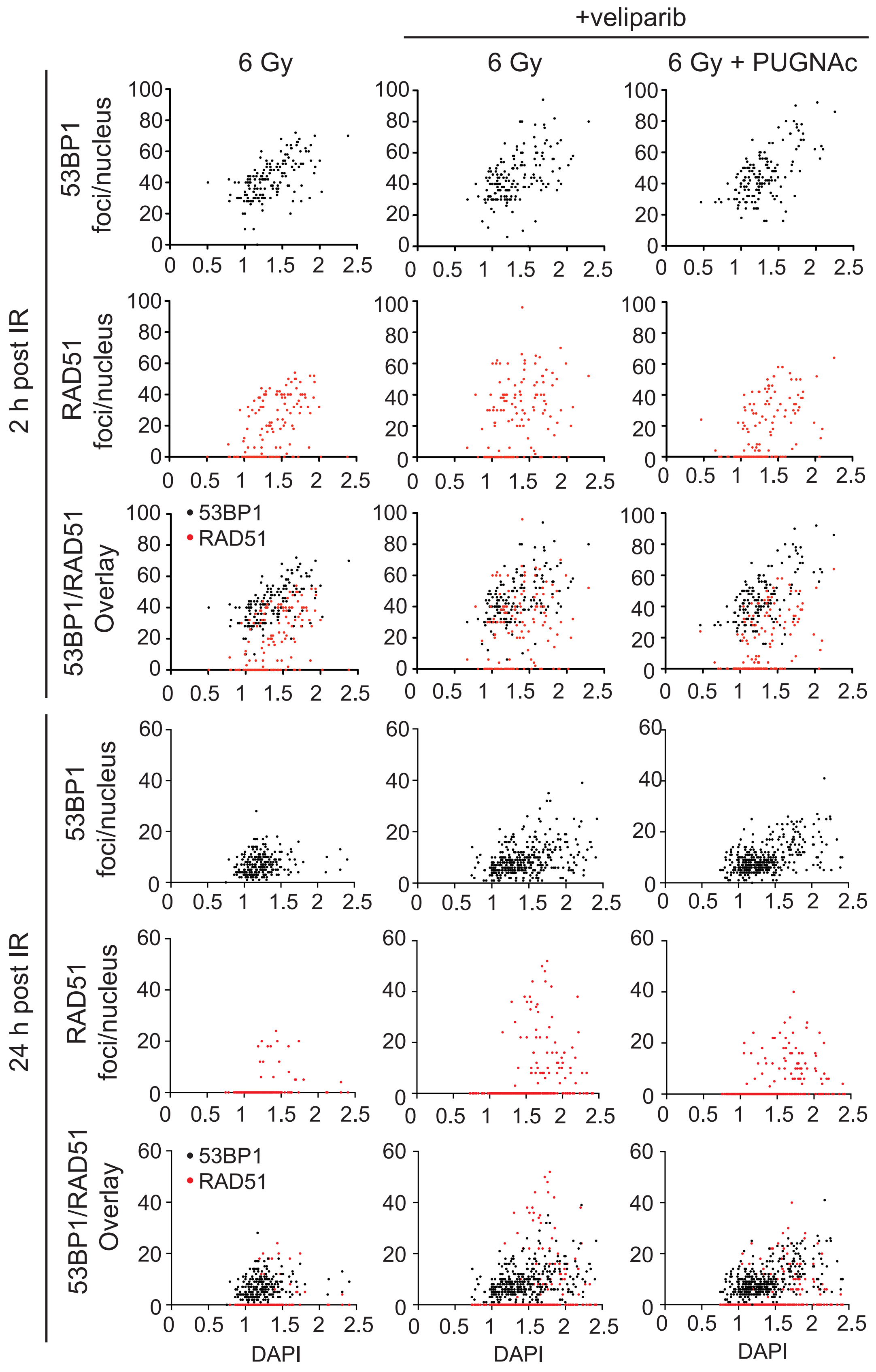

Fig. S6. O-GlcNAcylation antagonizes the effects of veliparib on the recruitment and retention of RAD51 after irradiation.

As also shown in **Fig. S5**, MCF7 cells were treated with DMSO or 50 µM PUGNAc ± 10 µM veliparib for 1 h prior to 6 Gy, then fixed after 2 h or 24 h and stained for 53BP1 and RAD51. Plots show 53BP1 (black) and RAD51 (red) foci per nucleus vs. normalized DAPI intensity for individual cells. Comparing the 2 h plots, reproduced from **Fig. S5C**, with the 24 h analysis indicates that increased O-GlcNAcylation by PUGNAc not only limits veliparib-induced recruitment (2 h) but also persistence (24 h) of RAD51 foci, consistent with its effects on BRCA1 recruitment and retention.

Journal of Cell Science • Supplementary information

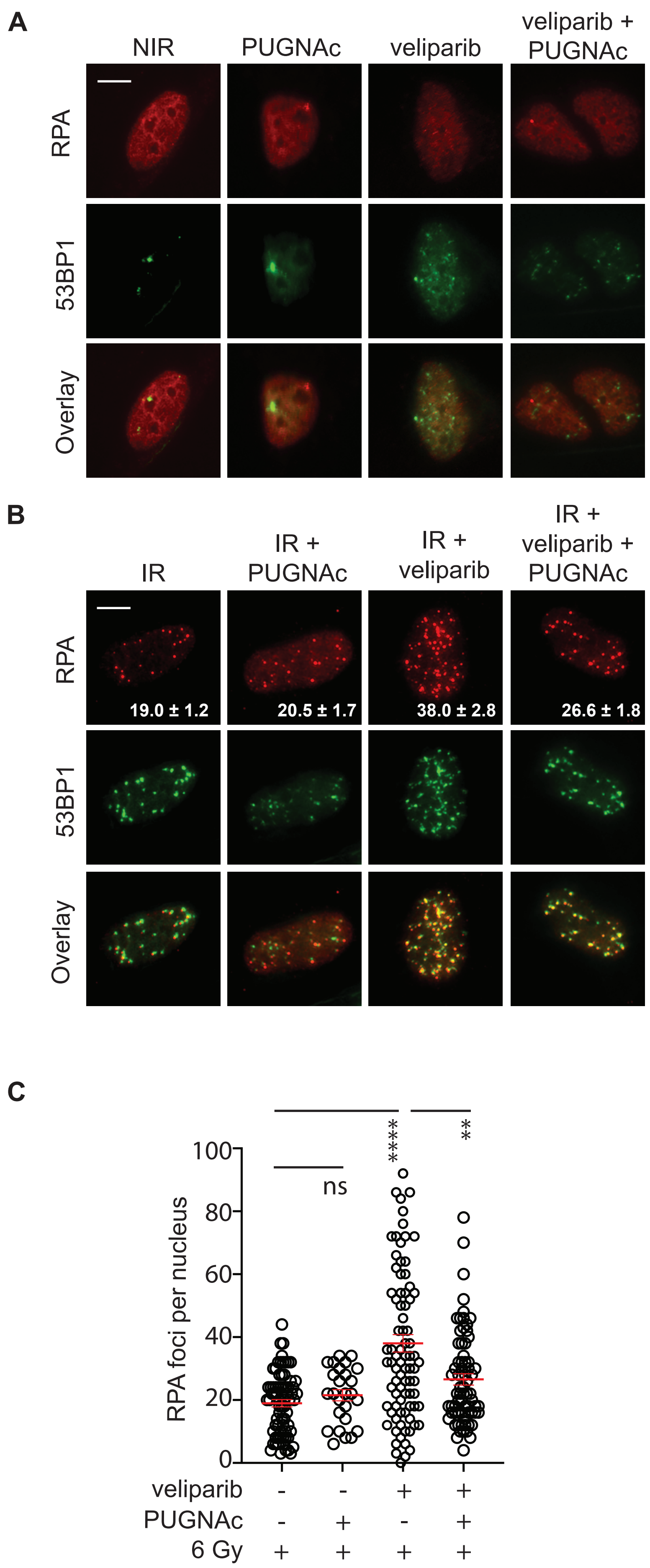

Fig. S7. PUGNAc antagonizes veliparib induced hyperresection after IR in HeLa cells
A and **B**. HeLa cells were treated for 1 h with DMSO vehicle, 50 μM PUGNAc, 10 μM veliparib, or 50 μM PUGNAc + 10 μM veliparib, followed by either no irradiation (**A**) or 6 Gy irradiation (**B**). Cells were fixed 24 h later and stained for RPA and 53BP1. Representative images show RPA foci in red with 53BP1 foci in green. Scale bar = 20 μm; inset is mean ± SEM foci per nucleus, n > 75. **C**. Quantification of RPA foci per nucleus. Red bars indicate mean ± SEM; n > 75. ****, $P < 0.0001$; ***, $P < 0.001$; **, $P < 0.01$; *, $P < 0.05$; ns, $P > 0.05$ (unpaired t-test).

Journal of Cell Science • Supplementary information

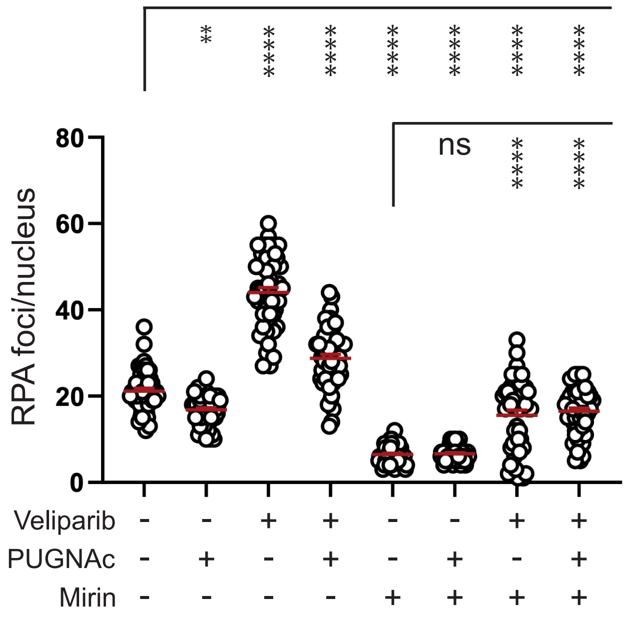

Fig. S8. Effects of veliparib ± PUGNAc on RPA foci formation upon irradiation depend on MRN-mediated DNA end resection.

MCF7 cells were treated for 1 h before 2 Gy with DMSO control, 50 µM PUGNAc, 10 µM veliparib, or PUGNAc + veliparib, with or without 50 µM mirin (an MRN complex inhibitor). Cells were then irradiated with 2 Gy and fixed after 3 h for RPA staining. Quantification of RPA foci per nucleus shows that mirin markedly reduced RPA foci under all conditions and suppressed the effects of PUGNAc, consistent with of MRN-dependent DNA end resection. Red bars indicate mean ± SEM; n > 50. ****, P < 0.0001; ***, P < 0.001; **, P < 0.01; *, P < 0.05; ns, P > 0.05 (unpaired t-test).

Journal of Cell Science • Supplementary information

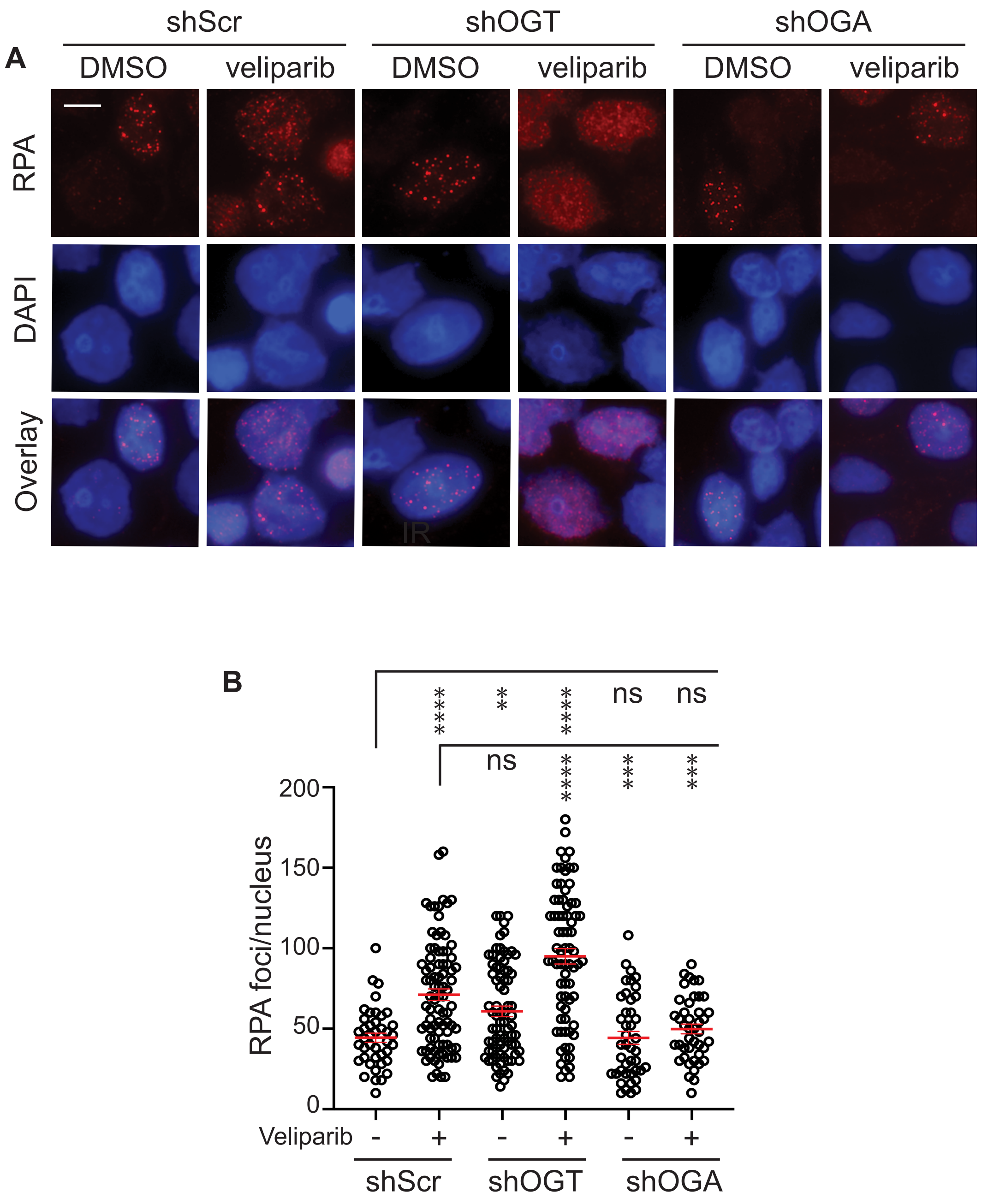

Fig. S9. Targeting OGT or OGA with shRNA affects veliparib-induced RPA foci formation after irradiation.

A. Expression of shScr, shOGT, and shOGA were induced for 48 h with 1 µg/ml doxycycline, and cells were treated with 0 or 10 µM veliparib for 1 h prior to 6 Gy, fixed after 2 h and stained for RPA. Scale bar = 20 µm. **B**. Quantification of RPA foci per nucleus, showing that shOGT promoted RPA foci formation alone and further enhanced the effects of veliparib, whereas shOGA attenuated veliparib effects. Red bars indicate mean ± SEM; n > 75. ****, $P < 0.0001$; ***, $P < 0.001$; **, $P < 0.01$; *, $P < 0.05$; ns, $P > 0.05$ (unpaired t-test).

Journal of Cell Science • Supplementary information

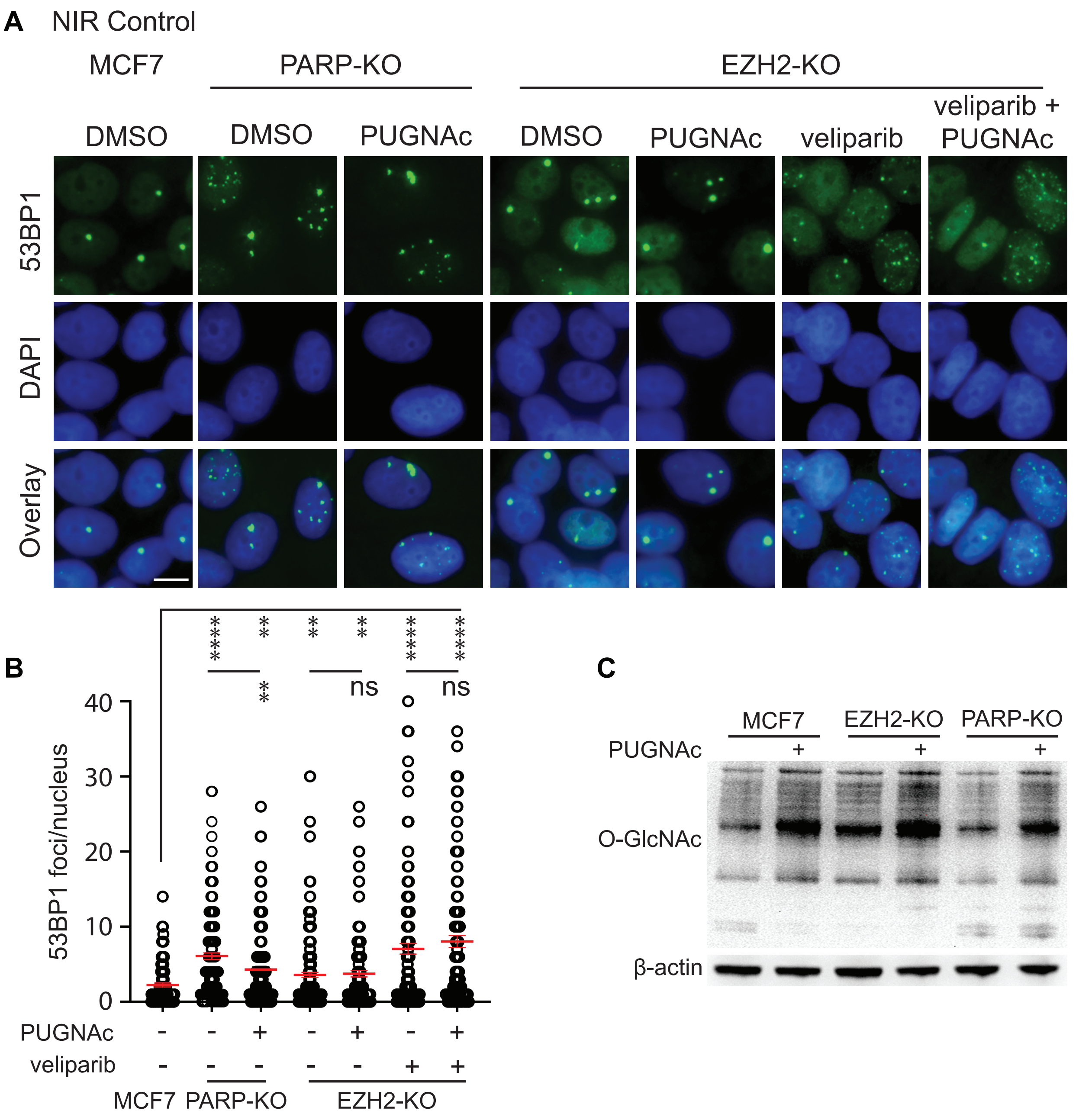

Fig. S10. PUGNAc increases O-GlcNAcylation and counteracts elevated 53BP1 foci in PARP1-KO but not in EZH2-KO cells under non-irradiated conditions.

A. Representative immunofluorescence images showing 53BP1 foci (green), DAPI stained DNA (blue), and their overlays in control MCF7, PARP1-KO or EZH2-KO cells treated with vehicle or the OGA inhibitor PUGNAc (50 µM) for 24 h. Scale bar = 20 µm. **B**. Quantification of 53BP1 foci per nucleus in individual cells from **A**, showing that PUGNAc reduced elevated 53BP1 foci in PARP1-KO cells but has no effect in EZH2-KO cells. Red bars indicate mean ± SEM; n > 70. ****, $P < 0.0001$; ***, $P < 0.001$; **, $P < 0.01$; *, $P < 0.05$; ns, $P > 0.05$ (unpaired t-test). **C.** Western blot showing O-GlcNAcylation levels increased after 24 h PUGNAc treatment (50 µM) in control MCF7, PARP1-KO, and EZH2-KO cells. Total cell lysates were analyzed, and β-actin was probed as a loading control.

Journal of Cell Science • Supplementary information

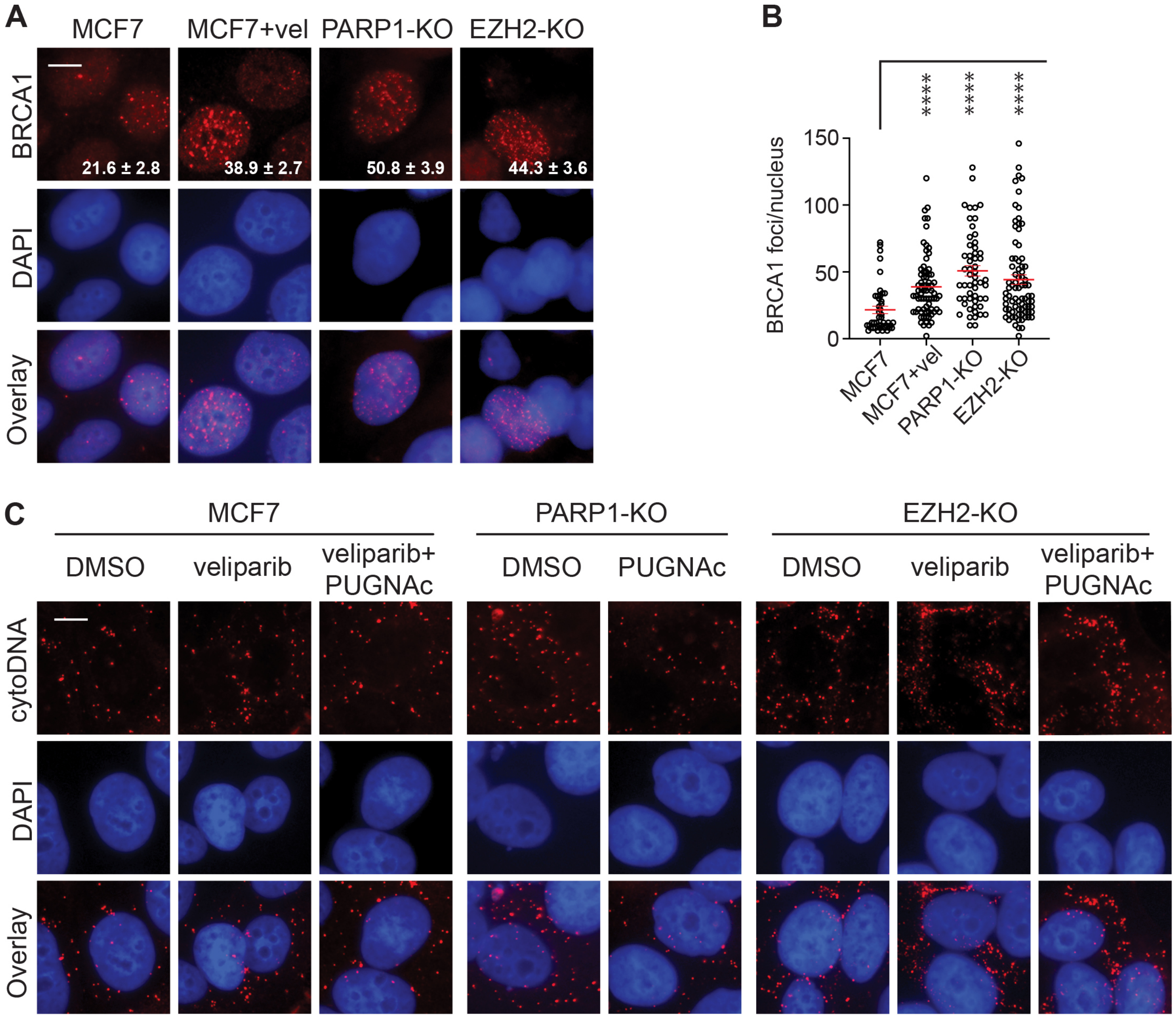

Fig. S11. Loss of EZH2 or PARP1 increases BRCA1 retention at DSBs after irradiation, while PUGNAc suppresses resection and cytosolic DNA accumulation in PARP1-KO but not in EZH2-KO cells.

A. MCF7 wild type (WT) cells, MCF7 WT pretreated with 10 µM veliparib for 1 h, PARP1-KO, and EZH2-KO cells were irradiated with 6 Gy, fixed 24 h post-irradiation, and stained with anti-BRCA1 antibody. Shown are BRCA1 foci (red), nuclear counterstain with DAPI (blue), and overlays. Inset is mean ± SEM foci per nucleus, n > 75. **B**. Plots of BRCA1 foci per nucleus for cells treated as in **A**, showing that loss of either PARP1 or EZH2 KO increased BRCA1 persistence. Red bars indicate mean ± SEM; n > 75. ****, P < 0.0001; ***, P < 0.001; **, P < 0.01; *, P < 0.05; ns, P > 0.05 (unpaired t-test). **C**. MCF7 WT, PARP1-KO, and EZH2-KO cells were treated with DMSO or 50 µM PUGNAc ± 10 µM veliparib for 1 h prior to 6 Gy. Cells were partially permeabilized and then fixed after 24 h stained with anti-DNA antibody to detect cytoplasmic DNA (cytoDNA) and counterstained with DAPI. Like the effects of PUGNAc on DNA end resection shown in Fig. 6, PUGNAc reduced cytoDNA accumulation in PARP1-inhibited or PARP1-KO cells, but not in EZH2-KO cells. For images, scale bar = 20 µm.

Journal of Cell Science • Supplementary information
