# Supplementary material for: Cancer metabolism in radiation sensitization: complementary roles of O-GlcNAc transferase (OGT) and PARP1
Source: J Cell Sci. Author manuscript; Available in PMC 2026 Mar 31. (PMC13036754; doi:10.1242/jcs.264322)
Supplement: Supplementary file 1 [file NIHMS2156830-supplement-1.pdf]

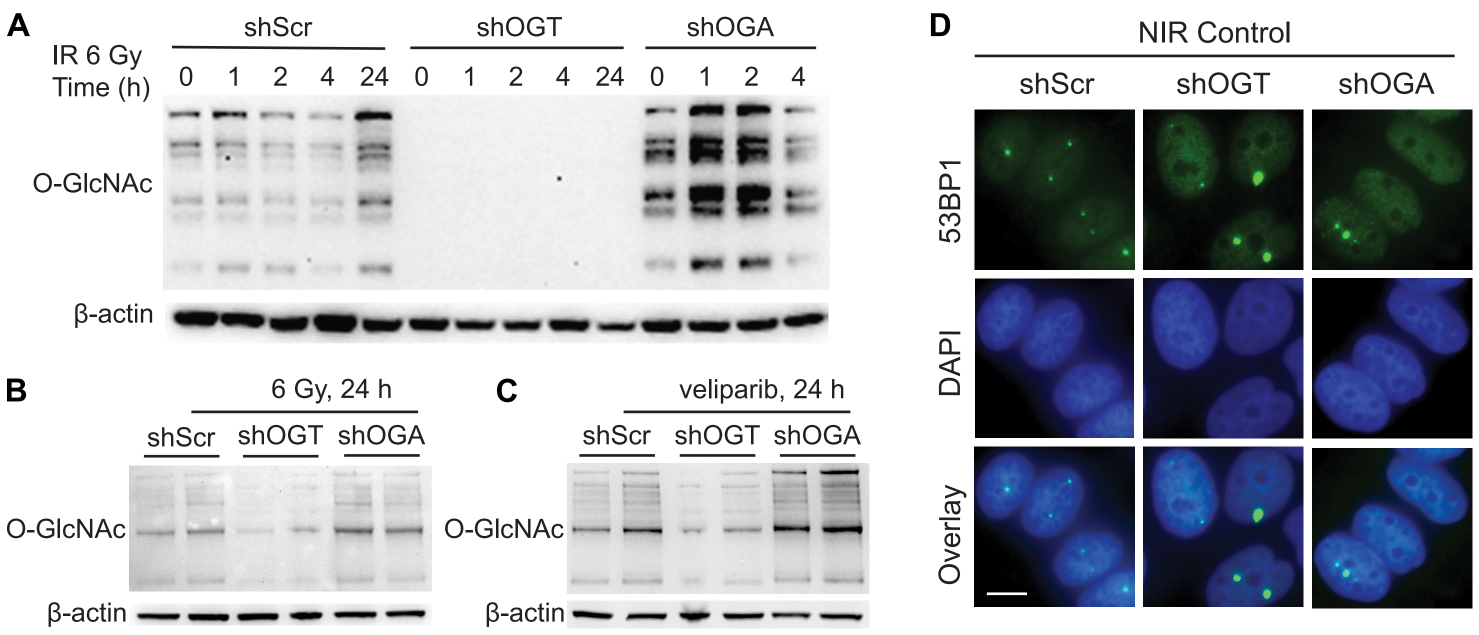


**Fig. S1. OGT and OGA knockdown modulate global O-GlcNAcylation without affecting baseline DNA damage foci.**

**A.** Western blot analysis showing the effects of OGT (shOGT) and OGA (shOGA) knockdown on global O-GlcNAcylation before and after 6 Gy. Cells expressing shScr, shOGT, or shOGA were treated with 1 μg/ml doxycycline for 48 h, irradiated with 6 Gy (IR), and harvested at the indicated time points. Non-irradiated (NIR) cells were collected at 0 h. **B-C**. Western blot showing O-GlcNAcylation levels following OGT or OGA knockdown in the presence or absence of 10 μM veliparib. shRNA expression was induced with 1 μg/ml doxycycline for 48 h. Then cells were harvested 24 h after 6 Gy or 10 μM veliparib. Non-irradiated or untreated shScr cells served as controls. For A-C, total cell lysates were analyzed, and β-actin was probed as a loading control. For panel C, the membrane shown in Fig. 1B was stripped of anti-OGT and subsequently reprobed to detect O-GlcNAcylation. **D**. Representative immunofluorescence images showing 53BP1 foci (pseudo-colored green), DAPI stained DNA (blue), and their overlays in shScr, shOGT, and shOGA cells under non-irradiated (NIR) conditions. Scale bar = 20 μm.

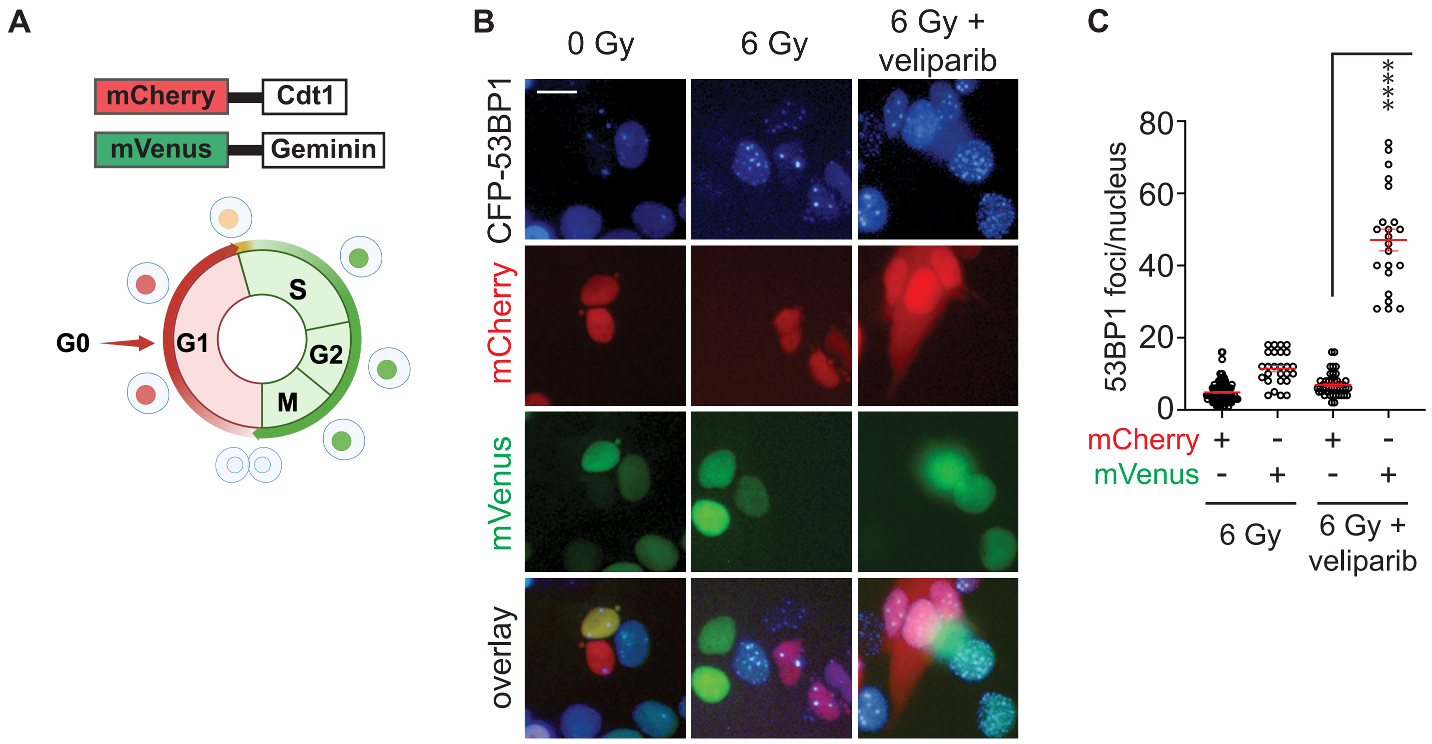


**Fig. S2. Veliparib induces foci persistence primarily in S/G2 cells after irradiation. A.** Schematic illustration of the FUCCI cell cycle reporter system. The G1 phase is characterized by increasing expression of mCherry-Cdt1 (red), the S/G2/M phase by mVenus-Geminin (green), and the G1/S transition by transient co-expression (yellow). **B**. MCF7 cells stably expressing FUCCI cell cycle reporters and 53BP1-CFP were treated with DMSO or 10 µM veliparib for 1 h prior to 6 Gy. After 24 h, cells were imaged by fluorescence microscopy to analyze 53BP1 foci (blue) and FUCCI reporters (red, green). Scale bar = 40 µm. C. 53BP1 foci in irradiated cells were quantified per nucleus and grouped by expression of FUCCI reporters, demonstrating that persistent 53BP1 foci were restricted to cells expressing mVenus-Geminin, suggesting a prolonged S/G2 phase associated with persistent DSBs. Red bars indicate mean ± SEM; ****, $P < 0.0001$ (unpaired t-test).

Journal of Cell Science • Supplementary information

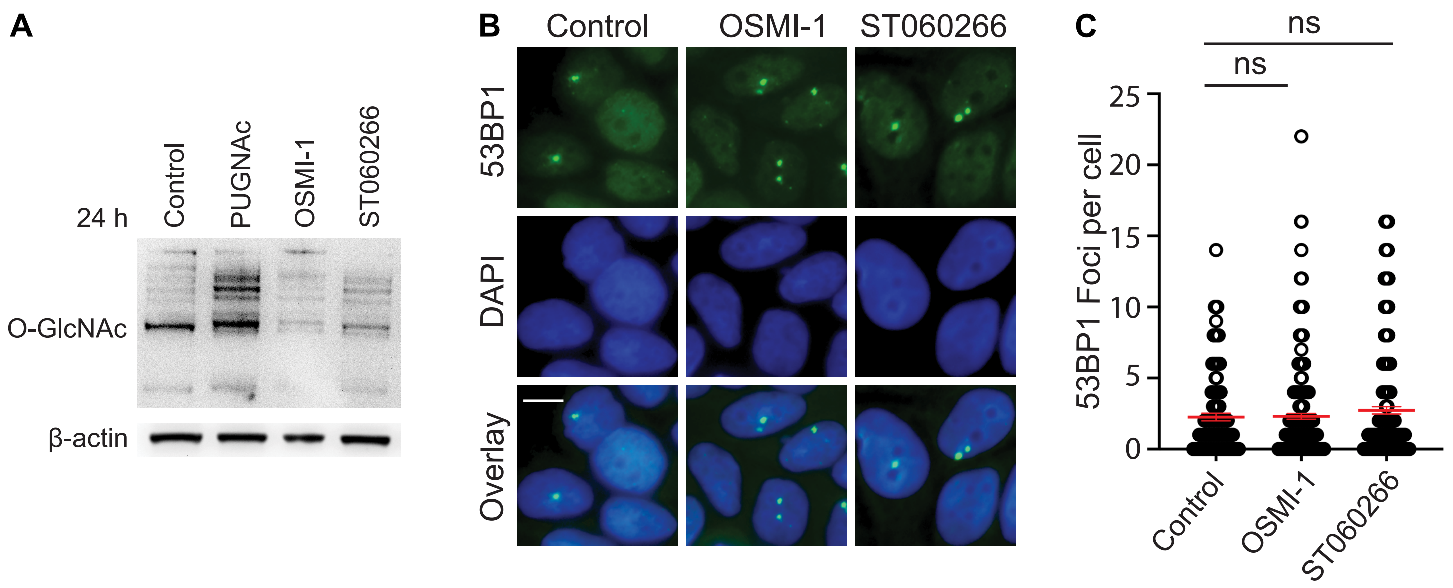


**Fig. S3. OGT inhibition reduces O-GlcNAcylation without affecting 53BP1 foci under non-irradiated conditions.**

**A.** Western blot showing O-GlcNAcylation levels after 24 h treatment with the OGA inhibitor PUGNAc (50 μM) or OGT inhibitors OSMI-1 (25 μM) or ST060266 (2.5 μM). Total cell lysates were analyzed, and β-actin was probed as a loading control. **B**. Representative immunofluorescence images showing 53BP1 foci (green), DAPI stained DNA (blue), and their overlays in MCF7 cells treated with vehicle control, OSMI-1 or ST060266 for 24 h. Scale bar = 20 μm. **C**. Quantification of 53BP1 foci per nucleus in individual cells from **B**, showing no effect of OGT inhibitors without irradiation. Red bars indicate mean ± SEM; ns, $P > 0.05$ (unpaired t-test).

Journal of Cell Science • Supplementary information

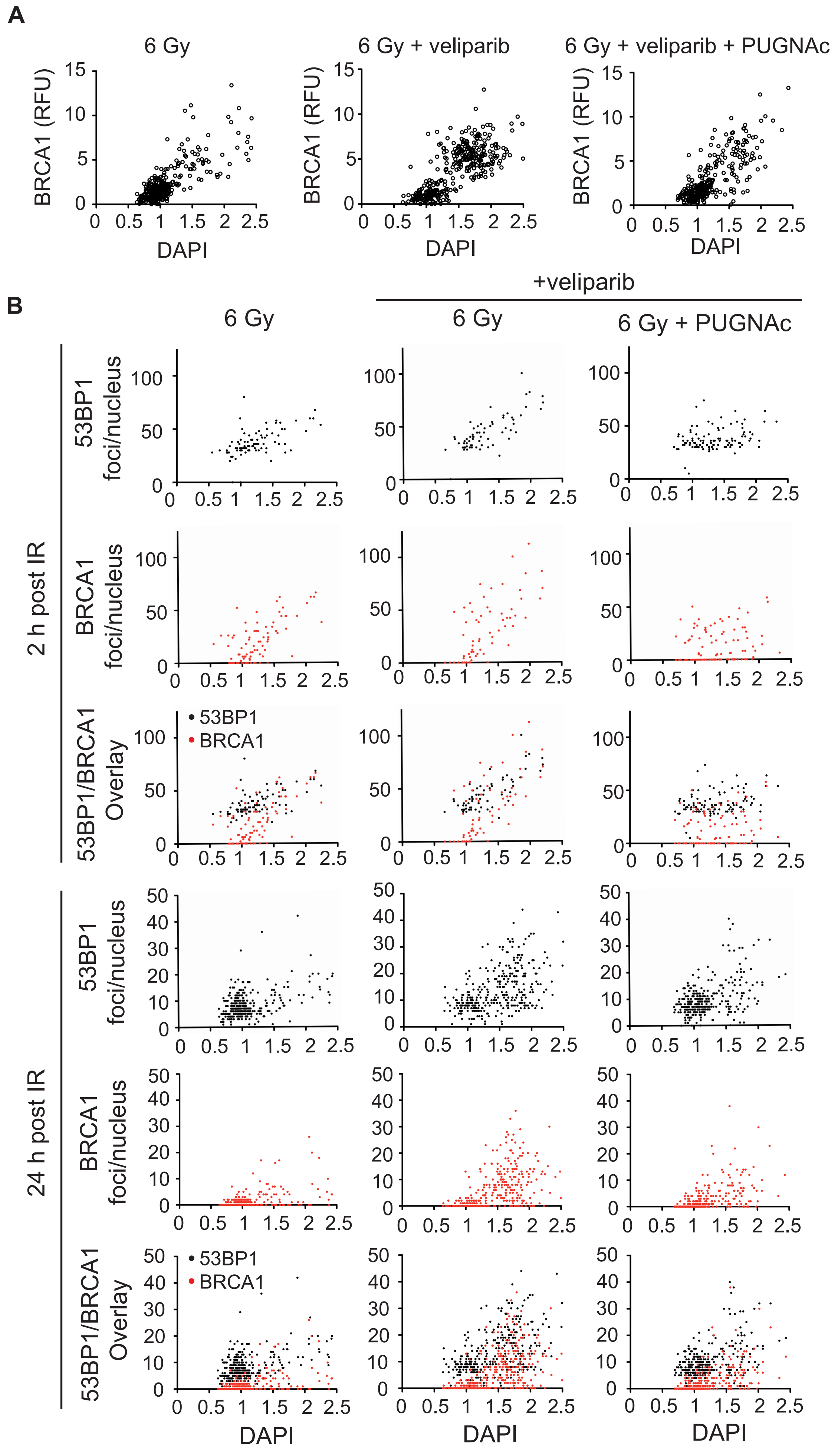


Journal of Cell Science • Supplementary information

**Fig. S4. O-GlcNAcylation antagonizes veliparib in the recruitment and retention of BRCA1 after irradiation.**

**A.** MCF7 cells were treated for 1 h prior to 6 Gy with 0 or 10 µM veliparib and DMSO or 50 µM PUGNAc, then fixed after 24 h and stained for BRCA1. To examine whether expression of BRCA1 may be disrupted in a way affecting detection of BRCA1 foci, total fluorescence intensity was quantified as relative fluorescence units (RFU) for individual nuclei and plotted vs. normalized DAPI intensity, indicating a similar expression pattern with respect to cell cycle stage. **B.** MCF7 cells were treated for 1 h prior to 6 Gy with 0 or 10 µM veliparib and DMSO or 50 µM PUGNAc, then fixed after 2 h or 24 h and stained for 53BP1 and BRCA1. Shown are plots of 53BP1 (black) and BRCA1 (red) foci per nucleus vs. normalized DAPI intensity.

Journal of Cell Science • Supplementary information

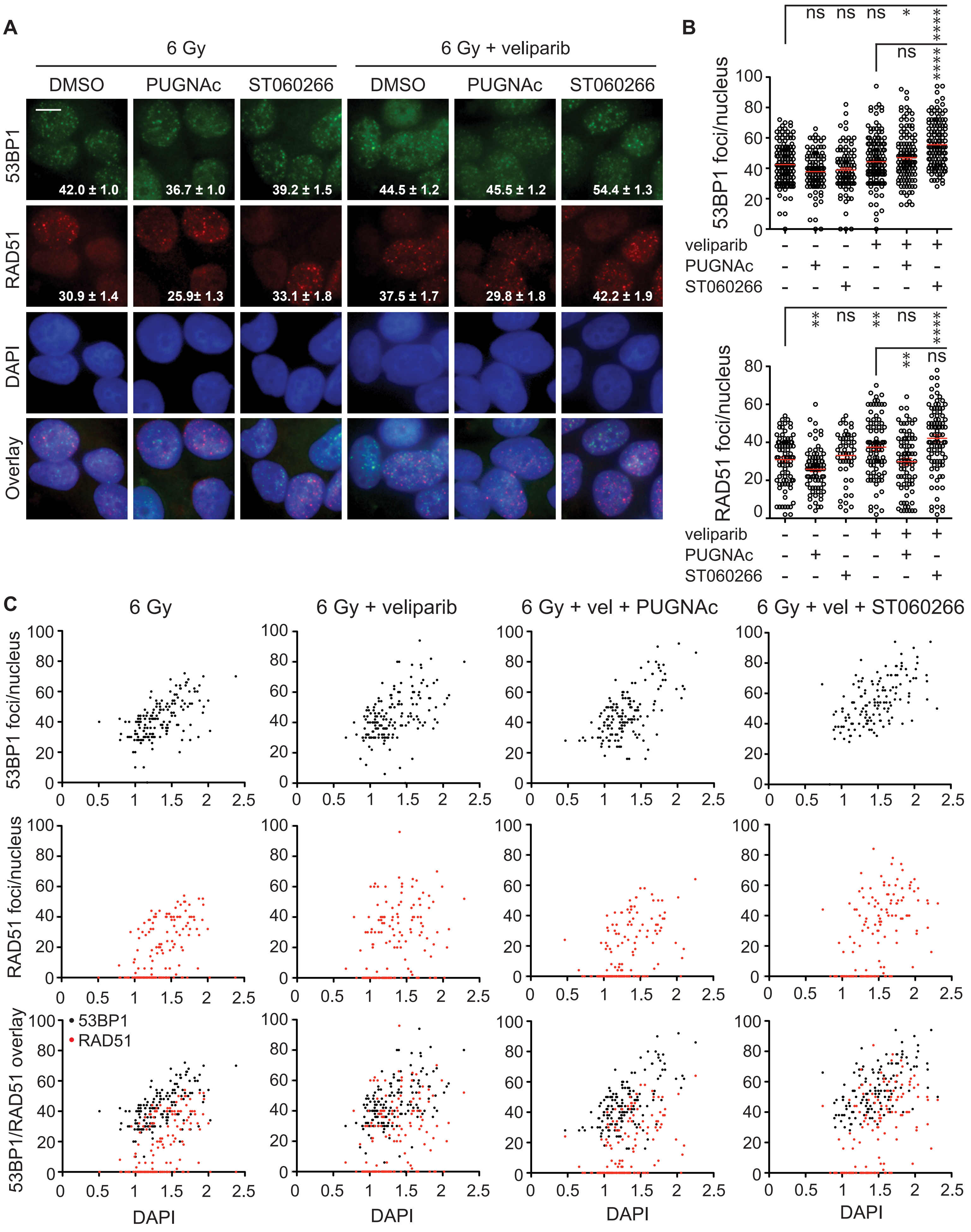


Journal of Cell Science • Supplementary information

**Fig. S5. OGT and OGA inhibitors differentially affect veliparib-induced RAD51 foci persistence after irradiation.**

**A**. Shown are representative images of MCF7 cells treated with DMSO, 50 μM PUGNAc or 2.5 μM OGT inhibitor ST0602660 ± 10 μM veliparib for 1 h prior to 6 Gy and then fixed after 2 h and stained for 53BP1 (green) and RAD51 (red) along with DAPI (blue). Scale bar = 20 μm. **B**. Quantification of 53BP1 and RAD51 foci per nucleus. Red bars indicate mean ± SEM; ****, $P < 0.0001$; ***, $P < 0.001$; **, $P < 0.01$; *, $P < 0.05$; ns, $P > 0.05$ (unpaired t-test). **C**. Shown are plots of 53BP1 (black) and RAD51 (red) foci per nucleus vs. normalized DAPI intensity.

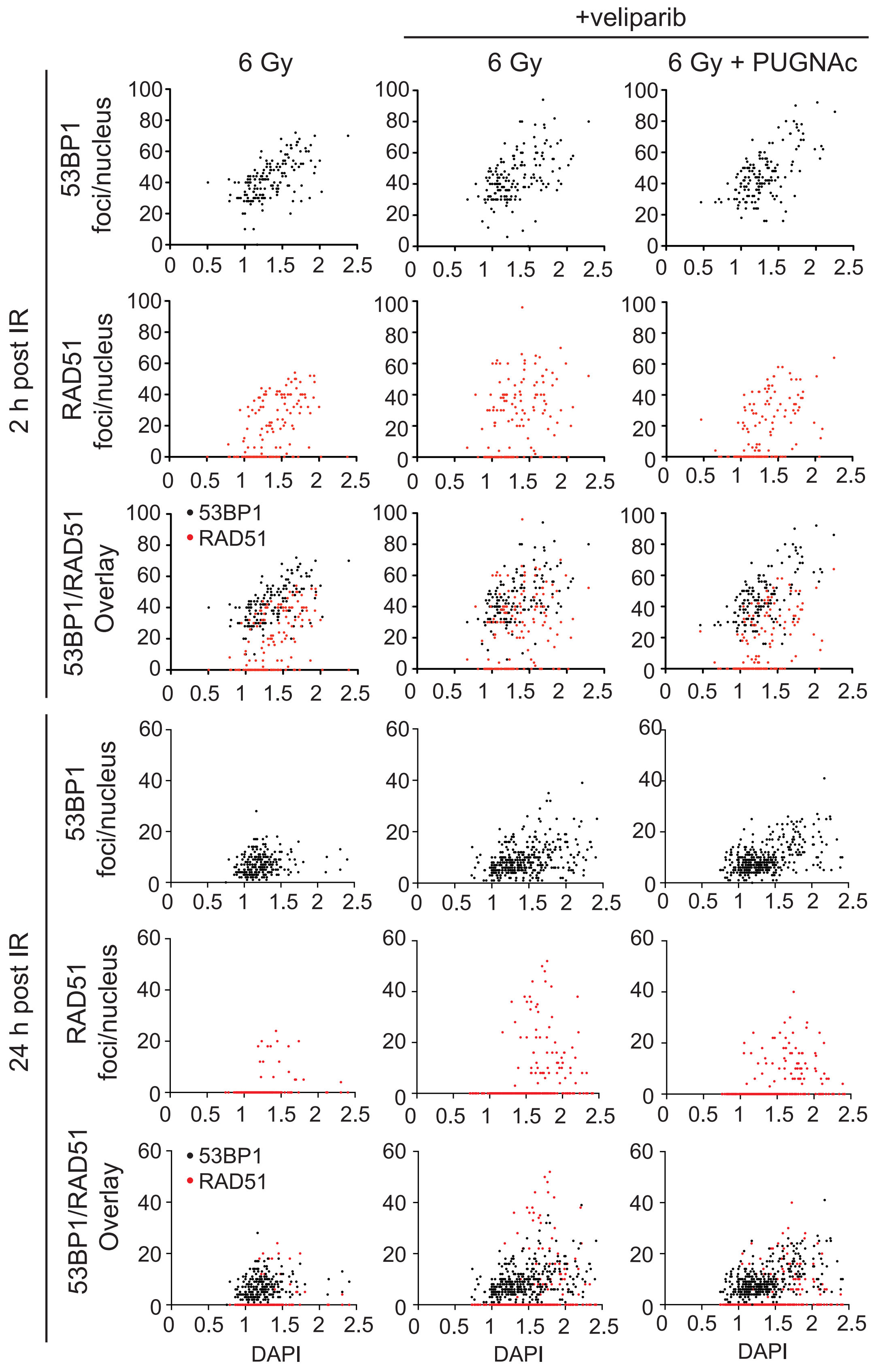


**Fig. S6. O-GlcNAcylation antagonizes veliparib in the recruitment and retention of RAD51 after irradiation.**

MCF7 cells were treated with DMSO or 50 μM PUGNAc ± 10 μM veliparib for 1 h prior to 6 Gy. Cells were fixed after 2 and 24 h and stained for 53BP1 and RAD51. Plots show 53BP1 (black) and RAD51 (red) foci per nucleus vs. normalized DAPI intensity, indicating that O-GlcNAcylation limits veliparib-induced recruitment (2 h) and persistence (24 h) of RAD51 foci.

Journal of Cell Science • Supplementary information

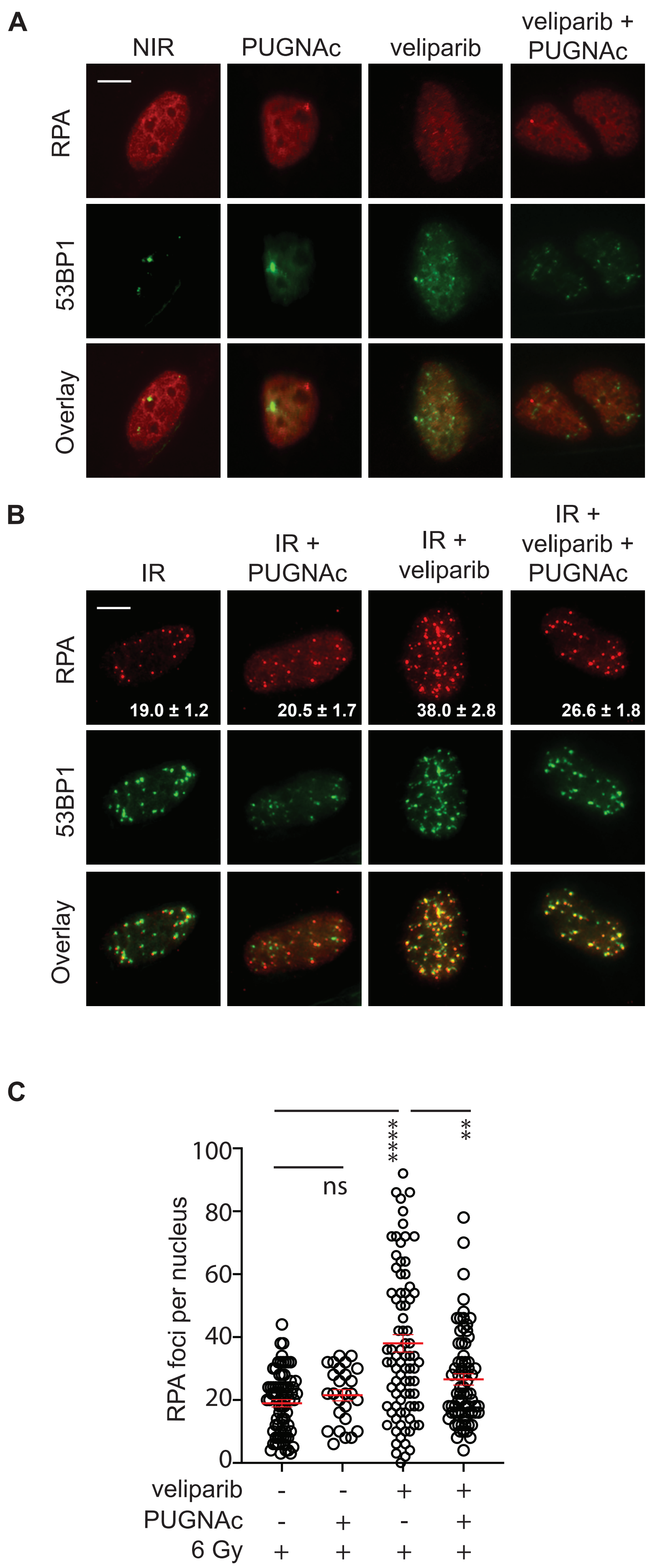


**Fig. S7. PUGNAc antagonizes veliparib induced hyperresection after IR in HeLa cells**

**A** and **B**. HeLa cells were treated for 1 h with DMSO vehicle, 50 μM PUGNAc, 10 μM veliparib, or 50 μM PUGNAc + 10 μM veliparib, followed by either no irradiation (**A**) or 6 Gy irradiation (**B**). Cells were fixed 24 h later and stained for RPA and 53BP1. Representative images show RPA foci in red with 53BP1 foci in green. Scale bar = 20 μm; inset is mean ± SEM foci per nucleus. **C**. Quantification of RPA foci per nucleus. Red bars indicate mean ± SEM; ****, $P < 0.0001$; ***, $P < 0.001$; **, $P < 0.01$; *, $P < 0.05$; ns, $P > 0.05$ (unpaired t-test).

Journal of Cell Science • Supplementary information

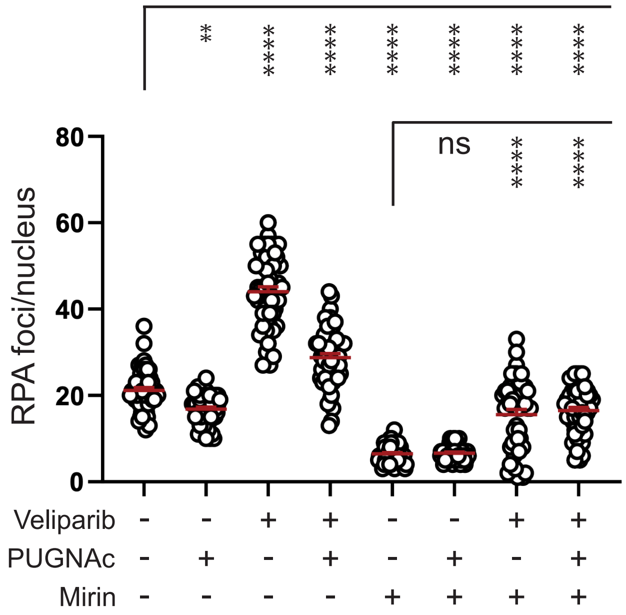


**Fig. S8. Veliparib ± PUGNAc on RPA foci formation upon irradiation via MRN- dependent DNA end resection**

MCF7 cells were treated for 1 h before 2 Gy with DMSO control, 50 μM PUGNAc, 10 μM veliparib, or PUGNAc + veliparib, with or without 50 μM Mirin (an MRN complex inhibitor). Cells were then irradiated with 2 Gy and fixed after 3 h for RPA staining. Shown are quantifications of RPA foci per nucleus. Red bars indicate mean ± SEM; ****, $P < 0.0001$; ***, $P < 0.001$; **, $P < 0.01$; *, $P < 0.05$; ns, $P > 0.05$ (unpaired t-test).

Journal of Cell Science • Supplementary information

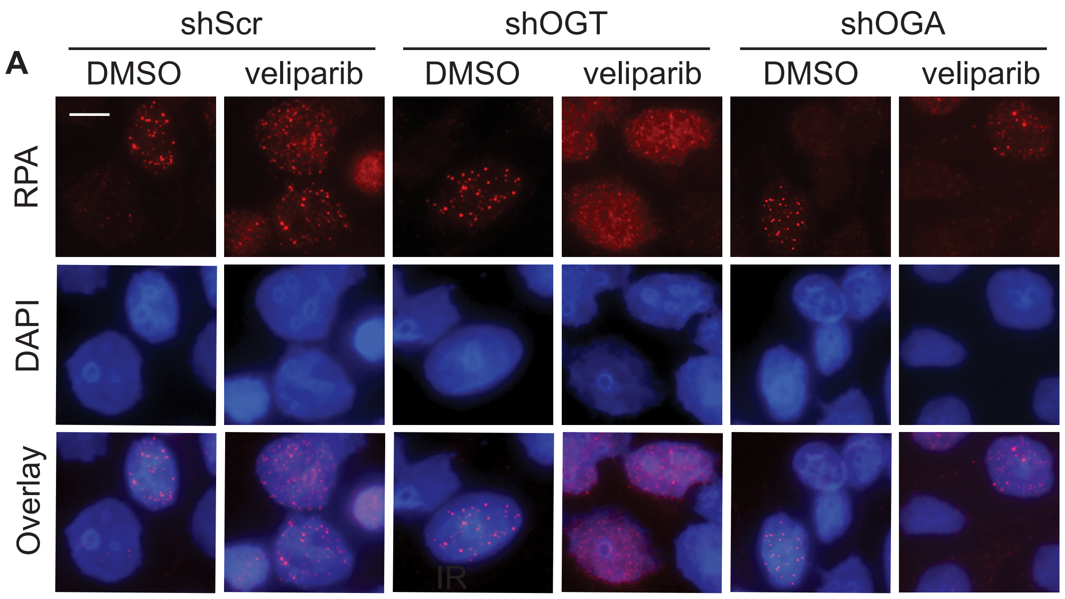


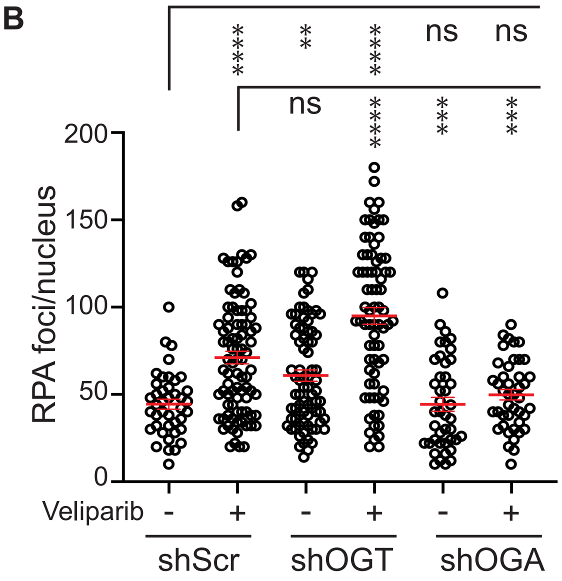


**Fig. S9. Targeting OGT or OGA with shRNA affects veliparib-induced RPA foci formation after irradiation.**

**A**. shScr, shOGT, and shOGA were induced for 48 h with 1 µg/ml doxycycline and cells were treated with 0 or 10 µM veliparib for 1 h prior to 6 Gy, fixed after 2 h and stained for RPA. Scale bar = 20 µm.

**B.** Quantification of RPA foci per nucleus. Red bars indicate mean ± SEM; ****, $P < 0.0001$; ***, $P < 0.001$; **, $P < 0.01$; *, $P < 0.05$; ns, $P > 0.05$ (unpaired t-test).

Journal of Cell Science • Supplementary information

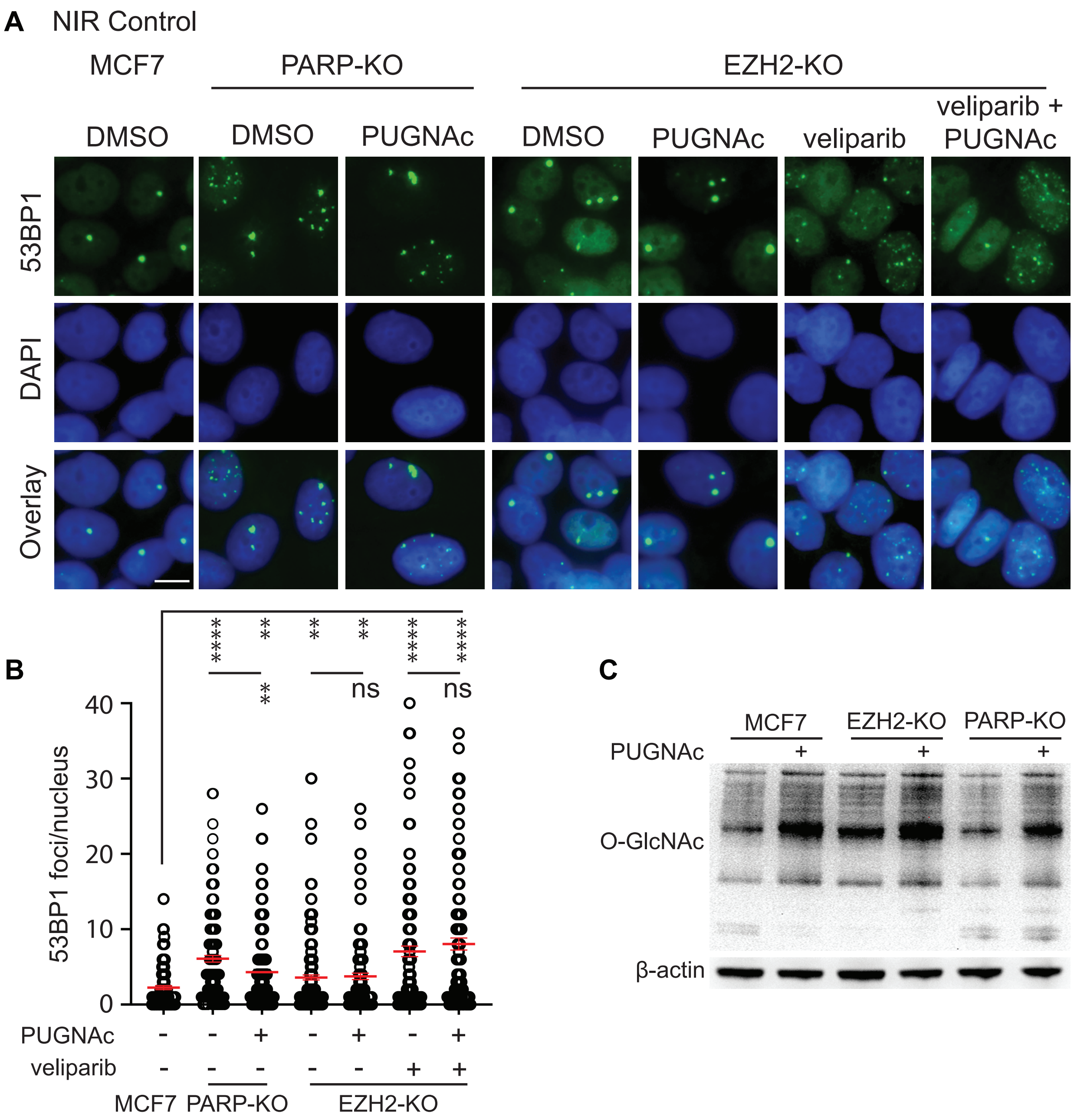


**Fig. S10. PUGNAc increases O-GlcNAcylation and counteracts elevated 53BP1 foci in PARP1-KO but not in EZH2-KO cells under non-irradiated conditions.**

**A.** Representative immunofluorescence images showing 53BP1 foci (green), DAPI stained DNA (blue), and their overlays in control MCF7, PARP1-KO or EZH2 KO cells treated with vehicle or the OGA inhibitor PUGNAc (50 μM) for 24 h. Scale bar = 20 μm. **B**. Quantification of 53BP1 foci per nucleus in individual cells from **A**, showing that PUGNAc reduces elevated 53BP1 foci in PARP1-KO cells but has no effect in EZH2-KO cells. Red bars indicate mean ± SEM; ****, $P < 0.0001$; ***, $P < 0.001$; **, $P < 0.01$; *, $P < 0.05$; ns, $P > 0.05$; ns, $P > 0.05$ (unpaired t-test). **C.** Western blot showing O-GlcNAcylation levels after 24 h PUGNAc treatment (50 μM) in control MCF7, PARP1-KO, and EZH2-KO cells. Total cell lysates were analyzed, and β-actin was probed as a loading control.

Journal of Cell Science • Supplementary information

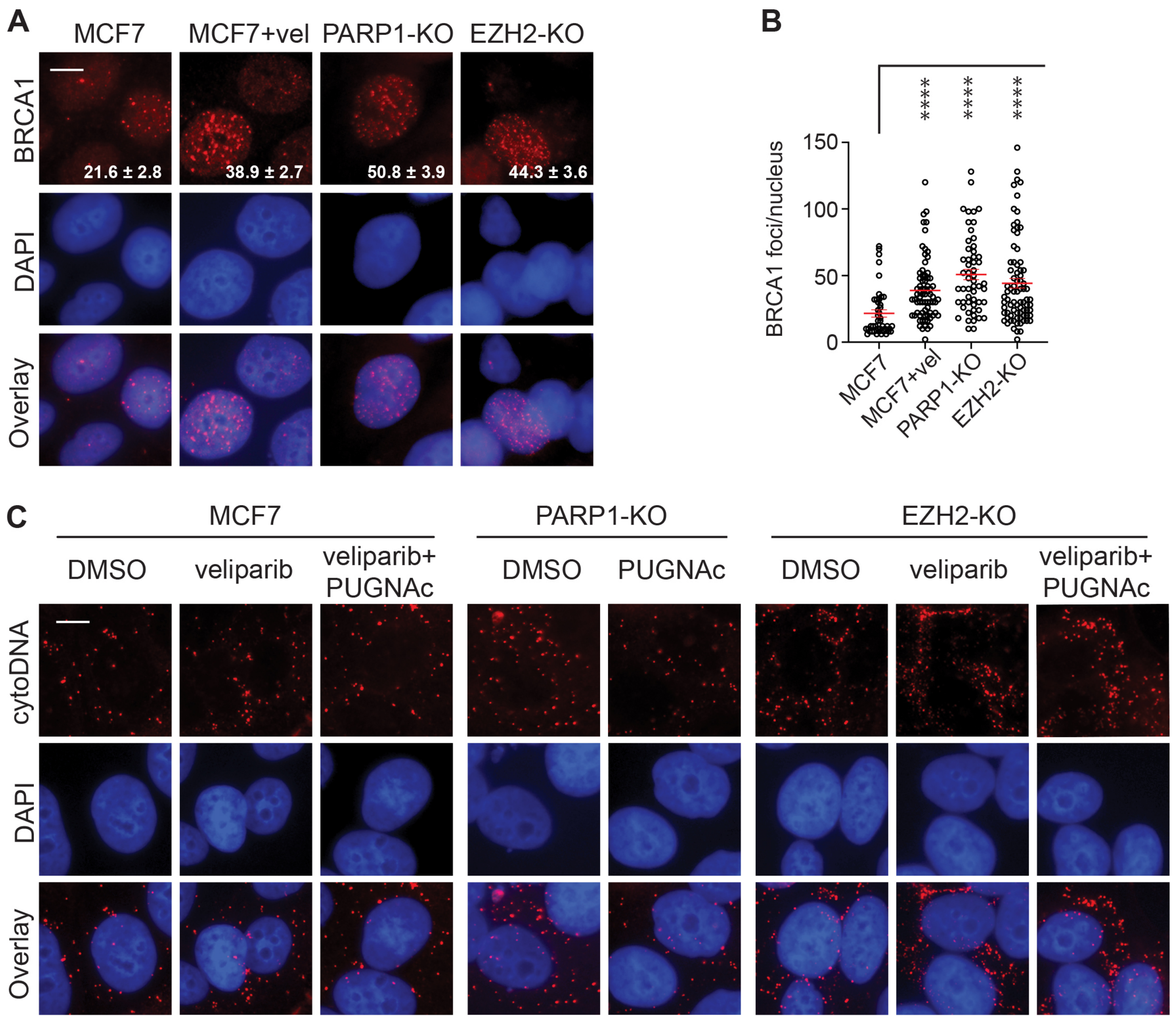


**Fig. S11. Loss of EZH2 or PAPR1 increases BRCA1 retention at DSBs, while PUGNAc suppresses resection and cytosolic DNA accumulation in PARP1- but not EZH2-knockout cells.**

**A**. MCF7 wild type (WT) cells, MCF7 WT pretreated with 10 μM veliparib for 1 h, PARP1-KO, and EZH2-KO cells were irradiated with 6 Gy, fixed 24 hours post-irradiation, and stained with anti-BRCA1 antibody. Shown are BRCA1 foci (red), nuclear counterstain with DAPI (blue), and overlays. Inset is mean ± SEM foci per nucleus. **B.** Plots of BRCA1 foci per nucleus for cells treated as in **A.** Red bars indicate mean ± SEM; ****, P < 0.0001; ***, P < 0.001; **, P < 0.01; *, P < 0.05; ns, P > 0.05 (unpaired t-test). **C.** MCF7 WT, PARP1-KO, and EZH2-KO cells were treated with DMSO or 50 μM PUGNAc ± 10 μM veliparib for 1 h prior to 6 Gy. Cells were partially permeabilized and then fixed after 24 h stained with anti-DNA antibody to detect cytoplasmic DNA (cytoDNA) and counterstained with DAPI. For images, scale bar = 20 μm.

Journal of Cell Science • Supplementary information
